# The Application of Artificial Intelligence to Cancer Research: A Comprehensive Guide

**DOI:** 10.1177/15330338241250324

**Published:** 2024-05-22

**Authors:** Amin Zadeh Shirazi, Morteza Tofighi, Alireza Gharavi, Guillermo A. Gomez

**Affiliations:** 1 Centre for Cancer Biology, 1067 SA Pathology and the University of South Australia, Adelaide, SA, Australia; 2 Department of Electrical Engineering, Faculty of Engineering, 68257 Bu-Ali Sina University, Hamedan, Iran; 3 Department of Computer Science, 125639 Azad University, Mashhad Branch, Mashhad, Iran

**Keywords:** cancer, artificial intelligence, machine learning, soft computing, machine vision, deep learning

## Abstract

Advancements in AI have notably changed cancer research, improving patient care by enhancing detection, survival prediction, and treatment efficacy. This review covers the role of Machine Learning, Soft Computing, and Deep Learning in oncology, explaining key concepts and algorithms (like SVM, Naïve Bayes, and CNN) in a clear, accessible manner. It aims to make AI advancements understandable to a broad audience, focusing on their application in diagnosing, classifying, and predicting various cancer types, thereby underlining AI's potential to better patient outcomes. Moreover, we present a tabular summary of the most significant advances from the literature, offering a time-saving resource for readers to grasp each study's main contributions. The remarkable benefits of AI-powered algorithms in cancer care underscore their potential for advancing cancer research and clinical practice. This review is a valuable resource for researchers and clinicians interested in the transformative implications of AI in cancer care.

## Artificial Intelligence Application to Cancer Research

**Figure 1.**
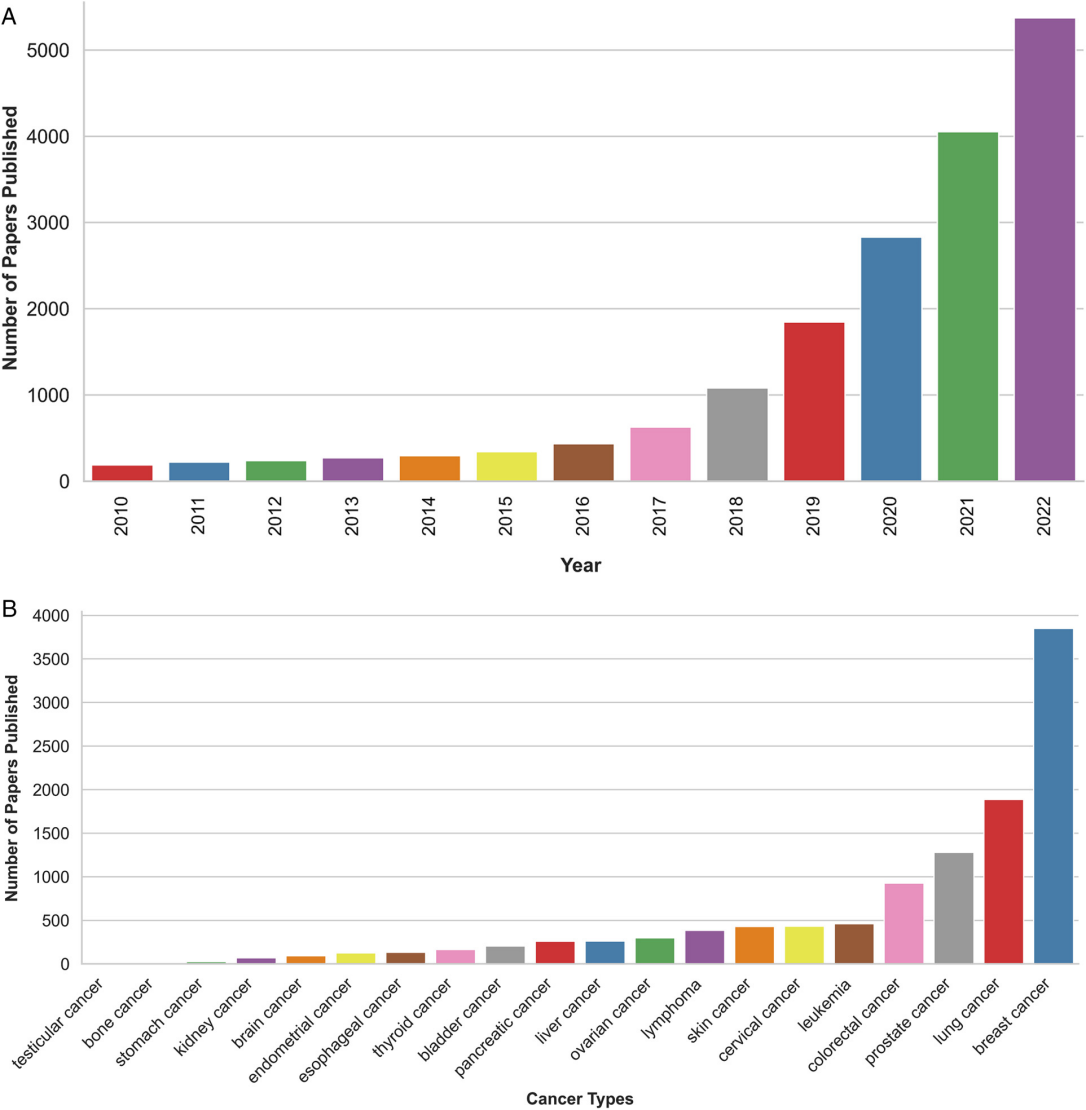
(A) The number of papers published in the field of “*AI applications to cancer research”* between 2010 and 2023 (keywords searched: “AI” and “Cancer”). (B) The number of AI-based models applied to different types of cancers between 2010 and 2022 (keywords searched: “AI” and “Cancer Type”). For A and B, the information was extracted from the PubMed database.^1^

Cancer continues to be a significant global health challenge, with early diagnosis, accurate prognosis, and personalized treatment being critical for improving patient outcomes. In recent years, the application of artificial intelligence (AI) has emerged as a promising approach to revolutionize cancer care, offering unprecedented opportunities for advancements in cancer research and clinical practice. Figure 1 shows the number of papers published on the application of AI to cancer research and the number of AI-based models applied to different cancers, highlighting the rapidly advancing field of AI in cancer research.

AI has shown promising results in studying different types of human cancers,^2–5^ including but not limited to cervical cancer,^6,7^ pancreatic cancer,^3,8^ breast cancer,^9,10^ colorectal cancer,^11^ ovarian cancer,^12^ laryngeal cancer,^13^ brain cancer,^14,15^ and lung cancer.^4,16,17^ In a scoping review, the extent of the use of AI and ML protocols for cancer diagnosis in prospective settings was explored.^2^ A literature review was also conducted on AI-driven digital cytology-based cervical cancer screening and highlighted the potential of this technology in resource-constrained settings.^6^ Another scoping review was conducted on the use of AI for the prediction and early diagnosis of pancreatic cancer, emphasizing the importance of early detection for improved survival rates.^3^ Meanwhile, a comprehensive overview of multifunctional magnetic nanostructures integrated with an AI approach for cancer diagnosis and therapy has been conducted.^18^ Indeed, a systematic review and meta-analysis have been conducted on the value of AI in lung cancer diagnosis, highlighting the potential of AI-assisted diagnosis in improving accuracy and efficiency.^4^ Another review was conducted on AI applications to find the most critical features extracted from brain cancer patients’ MRI, histopathology, and CT scan images.^19^ These studies have shown promising results in improving cancer diagnosis accuracy, sensitivity, and specificity, potentially reducing the burden on healthcare providers and improving patient outcomes. Furthermore, AI has been integrated with other technologies, such as radionics and genomics, to enhance cancer diagnosis and prediction.^20,21^

**Figure 2.**
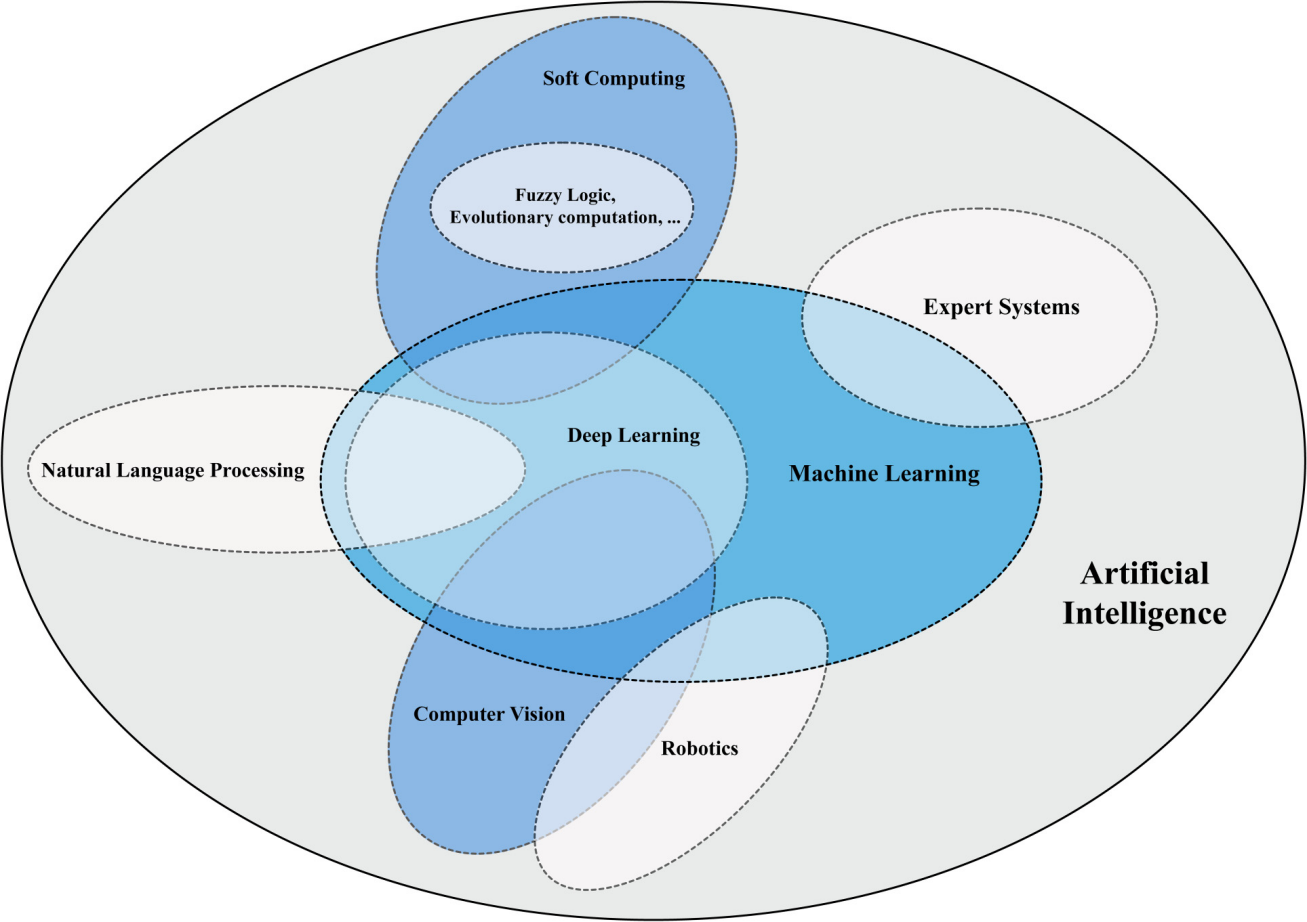
AI subfields and the strategies applied to cancer research in this study. Blue areas emphasize the focus of this review.

This paper reviews AI's role in cancer diagnosis, covering its current use, challenges, and future directions. It focuses on how machine learning, deep learning, and soft computing, illustrated in Figure 2, enhance cancer research by aiding in early detection, accurate diagnosis, treatment prediction, and monitoring tumor recurrence. The aim is to give a thorough overview of AI's application in oncology and demonstrate how these AI subfields can boost diagnostic precision, efficiency, and patient care, with detailed exploration of their applications.

### Machine Learning in Cancer Research

Machine learning, a subfield of AI, has been extensively applied in cancer research because of its ability to analyze complex data patterns and make accurate predictions. Several studies have reviewed the application of machine learning in cancer research, providing valuable insights into its potential and limitations.^22–24^

As a machine learning method, decision trees have demonstrated numerous applications in medicine and public health, particularly in addressing various issues within the field of cancer.^25–27^ Such applications span an array of cancer types, including breast,^28–32^ gastric,^33,34^ thyroid,^35^ prostate,^36^ and colorectal cancer.^37^ These studies have exhibited significant improvements in the accuracy of cancer diagnoses.^30,33^ Additionally, the k-means machine learning algorithm has been employed in several types of cancer, such as breast^38,39^ and skin cancer.^40^ Another technique, K-nearest neighbors (KNN), has also been used in cancer research,^41^ yielding enhanced accuracy in cancer prediction.^41^ Furthermore, logistic regression is a widely used technique in cancer research,^42^ aiding in the improvement of various cancer diagnoses, including gastric,^43^ colon,^44^ and bladder cancer,^45^ among others. Naïve Bayes, another powerful machine learning technique, has also found applications in the field of cancer.^46,47^ Principal component analysis has also proven valuable for various cancer types, such as lung,^48^ cervical,^49^ and colorectal cancer.^50^ Random Forests, an ensemble machine learning method, has been employed in numerous cancer diagnoses, including breast,^51,52^ ovarian,^53^ thyroid,^54^ and cervical cancer.^55^ Lastly, eXtreme Gradient Boost, a classification machine learning technique, has been applied to different types of cancer, such as gastric cancer.^56^

### Deep Learning in Cancer Research

Deep learning, another subfield of AI, has gained significant attention in cancer research because of its ability to automatically learn complex features from large datasets, making it particularly suitable for tasks such as image analysis and molecular profiling. Several studies have reviewed the application of deep learning in cancer research, highlighting its potential in advancing cancer diagnosis, prognosis, and treatment prediction.^57,58^

In addition, a large-scale synthetic pathological image dataset called SNOW for breast cancer research, which aids in computational pathology by providing diverse data for deep learning model training, was introduced.^59^ A hybrid technique combining deep learning architectures with machine learning classifiers and fuzzy min–max neural networks for cervical cancer diagnosis, achieving high classification accuracy in Pap smear image classification, was proposed by Kalbhor et al.^60^ Meanwhile, a deep-learning-based method was developed to automatically identify circulating tumor cells and cancer-associated fibroblasts, with significantly better results than conventional computer vision methods.^61^ In addition, an enhanced breast histopathology image analysis for cancer detection using variational autoencoders and convolutional neural networks (CNNs) was proposed, resulting in improved prediction accuracy.^62^

### Soft Computing in Cancer Research

**Figure 3.**
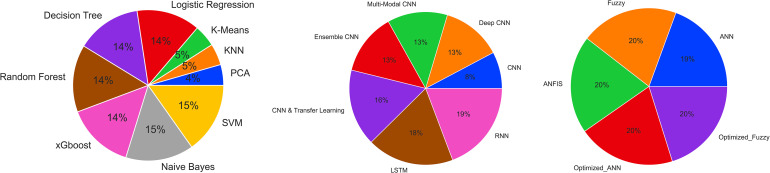
Frequency of algorithms used in different AI subfields in cancer research (obtained from PubMed database^1^ between 2010 and 2023).

Soft computing, a subfield of AI encompassing fuzzy logic and evolutionary algorithms, has also been widely applied in cancer research, offering unique advantages in dealing with uncertainty and imprecision in cancer data. A hybrid approach using the cuckoo search (CS) algorithm and artificial bee colony (ABC) for optimizing feature selection in microarray data for cancer classification was developed.^63^ The proposed method improved the performance of the Naïve Bayes classifier and provided better results than other feature selection algorithms. In addition, in the work of Gumaei et al,^64^ a method for prostate cancer diagnosis using correlation feature selection (CFS) and random committee (RC) ensemble learning was proposed. The technique achieved a 95.098% accuracy rate, outperforming other methods on the same dataset. Indeed, a computer-aided diagnosis system for skin cancer based on soft computing techniques, including CNNs and satin Bowerbird optimization (SBO), was introduced in the work of Xu et al.^65^ The system demonstrated high accuracy, sensitivity, and specificity compared with other methods in the literature. A combined approach to bilevel feature selection techniques using soft computing methods for classifying colon cancer was proposed in the work of Prabhakar et al.^66^ The method combined the multivariate minimum redundancy–maximum relevance (MRMR) technique with optimization techniques such as invasive weed optimization (IWO) and teaching learning-based optimization (TLBO). The approach achieved a classification accuracy of 99.16% when using quadratic discriminant analysis (QDA). A medical decision support system for assessing gastric cancer risk factors based on fuzzy cognitive maps (FCMs) was presented in the work of Mahmoodi et al.^67^ The system showed higher accuracy than other decision-making algorithms, including decision trees, Naïve Bayes, and artificial neural network (ANN). In addition, a cloud-based breast cancer prediction system that uses soft computing approaches, including Type-1 fuzzy logic (T1F) and SVM, was constructed.^68^ The system achieved a higher precision rate in breast cancer detection than other methods, with BCP-SVM providing 97.06% accuracy. Figure 3 shows the frequency of the use of machine learning, deep learning, and soft computing algorithms in cancer research between 2010 and 2023.

**Figure 4.**
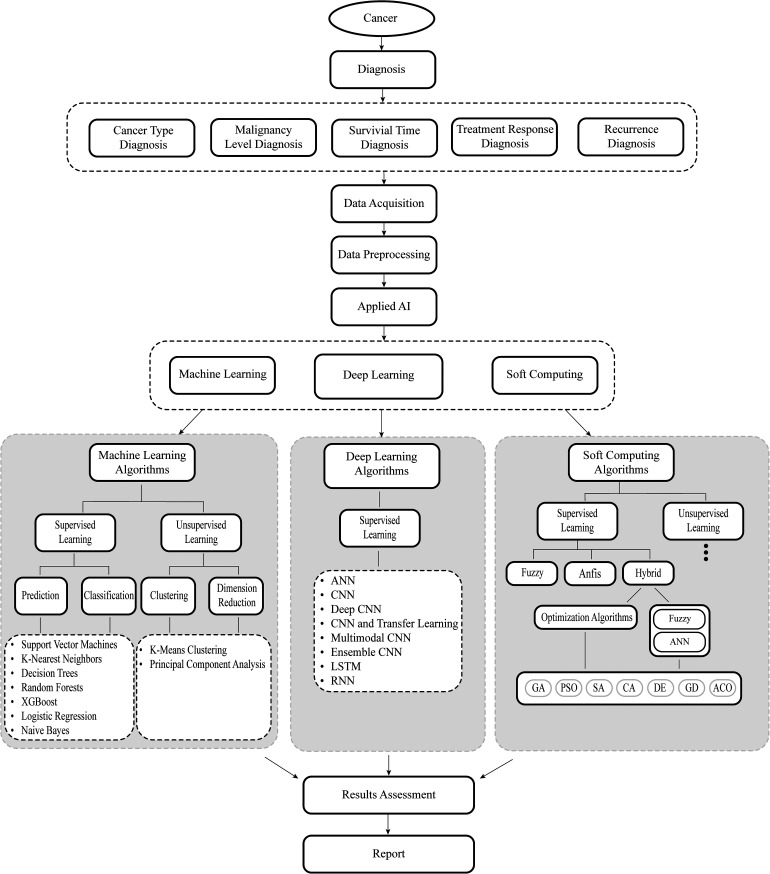
The workflow of each article investigated in this article (AI: artificial intelligence, XGBoost: eXtreme Gradient Boost, ANN: artificial neural network, CNN: convolutional neural network, LSTM: long short-term memory networks, RNN: recurrent neural network, Anfis: adaptive network-based fuzzy inference system).

The section details the key algorithms and performance metrics in Machine Learning, Soft Computing, and deep learning, with a systematic overview of studies using these methods in cancer research presented in a table (referenced as Figure 4). It also discusses the challenges and prospects of AI in oncology. The aim was to offer an impartial, informative view on the current AI applications in this field, tailored for a wide audience including researchers and clinicians from various disciplines, without giving personal opinions on individual studies due to space constraints. This approach seeks to provide a clear, objective insight into the extensive application and potential of AI in cancer research.

### Performance Metrics

Below, we indicate the formulas for several quantitative measures used to evaluate the performance of the proposed framework in the literature, including accuracy, sensitivity, specificity, precision, recall, F1-score, Matthews's correlation coefficient (MCC), receiver operating characteristic (ROC) curve, and area under the curve (AUC).

(1) $$Accuracy=\frac{TP+TN}{TP+FP+FN+TN}$$

(2) $$Sensitivity=\frac{TP}{TP+FN}$$

(3) $$specificity=\frac{TN}{TN+FP}$$

(4) $$\mathrm{Pr}ecision=\frac{TP}{TP+FP}$$

(5) Recall=TPTP+FN

(6) F1−Score=2×Precision×RecallPrecision+Recall

(7) $$MCC=\frac{TP\times TN-FP\times FN}{\sqrt{\left(TP+FP)(TP+FN)(TN+FP)(TN+FN\right)}}$$

(8) $$AUC=\frac{S_{p}\text{-}n_{p}(n_{n}+1)/2}{n_{p}\times n_{n}}$$

where TP, TN, FP, and FN denote true positives, false negatives, and false negatives, respectively. These values are derived from the confusion matrix. For AUC, *Sp_p_* denotes the sum of the ranks of all positive samples, and *n_p_* and *n_n_* denote the number of positive and negative samples, respectively.

### Pre-Processing

The preprocessing step is crucial in preparing the raw data and making it compatible with the machine-learning problem. The main objective of preprocessing is to ensure the model's convergence and improve its training speed. Various techniques and tools are used to preprocess data, including the following steps.

1. ***Data cleaning*** mainly involves handling missing values, checking for outliers and inconsistent data, eliminating errors, and transforming the data into a uniform format.
2. ***Data transformation*** is required when attributes are of different types (structured data, medical images, free text, etc). It converts it to a form that is understandable and acceptable for machine learning models.
3. ***Dimensionality reduction*** is used to reduce the dimensionality of a dataset having extensive features or variables, leading to a less complex architecture and preventing overfitting issues.
4. ***Data normalization*** is used to maintain the general distribution in the dataset, ensure the model's convergence, and improve the model's training speed.
5. ***Feature extraction*** produces new low-dimensional features that represent the original high-dimensional features. This is done by identifying the important features or patterns of the data.
6. ***RGB to grayscale conversion*** is used to convert input RGB images to grayscale images.
7. ***Data augmentation*** is a strategy used to extend the data with a set of transformations of the original data, helping overcome overfitting when training on very little data.
8. ***Image quality enhancement*** is used to improve the visibility of features in an image by changing its attributes, such as contrast and brightness.
9. ***Image denoising*** is used to restore an image to its original quality by reducing or removing noise to guarantee accurate information analysis while preserving vital details such as edges and texture information.
10. ***Image filtering*** is a technique to remove unwanted features from images, such as noise, bubbles, and hair.
11. ***Image segmentation*** is used to divide an image into several segments or objects, which is a crucial stage in computer vision.

## Machine Learning Application to Cancer Research

This section explores machine learning algorithms like SVM, Naïve Bayes, Decision Trees, Random Forests, K-means, KNN, Logistic Regression, eXtreme gradient boost (XGBoost), and Hybrid models within cancer research. It examines how these algorithms, with their distinct advantages in accuracy, adaptability, or scalability, analyze vast datasets to uncover new biomarkers, craft personalized treatments, and enhance early detection, ultimately improving patient outcomes. The discussion will detail each algorithm's features, strengths, and specific uses in oncology.

### Support Vector Machines

**Figure 5.**
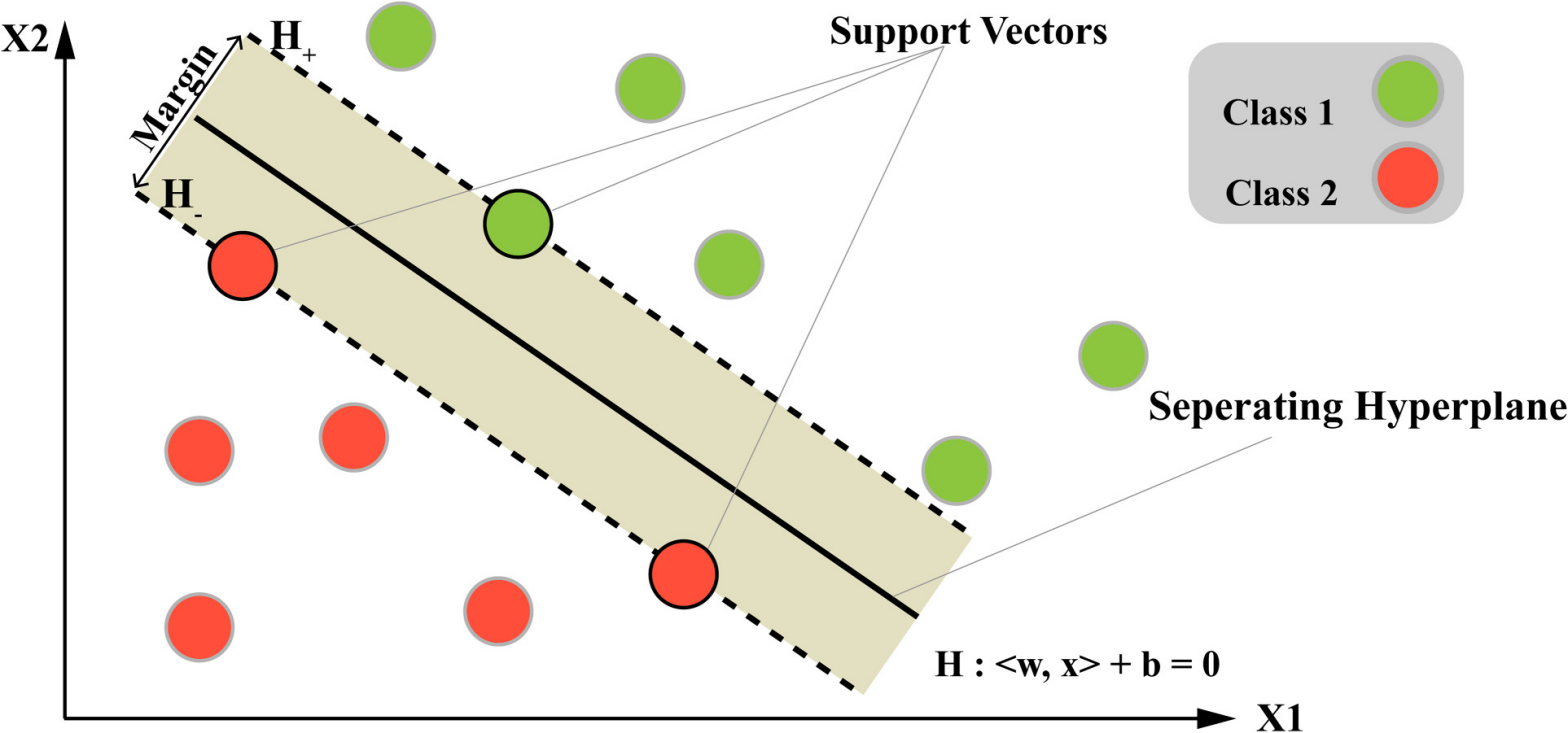
Structure of the support vector machine (SVM) algorithm.

SVM is a machine learning methodology that essentially functions as a mechanism for sorting data into separate categories by determining the optimal partition or border between them. In the realm of cancer research, SVM serves as a tool for classification, such as differentiating malignant (class 1) from benign tumors (class 2), as shown in Figure 5. Consider a dataset with information on tumor size, shape, and texture collected from many patients. Based on these attributes, the SVM model can use these data to identify the most suitable demarcation between malignant and benign tumors. Based on these features, the SVM algorithm finds a decision boundary (separating hyperplane) that separates malignant and benign tumors in a multi-dimensional space. The support vectors are the tumors closest to this boundary, which “support” the position of the hyperplane. The margin is the distance between the hyperplane and support vectors, which the SVM algorithm seeks to maximize for a more accurate classification. Once the model is trained, it can predict tumor classification for new patients, thus empowering physicians to make more well-informed choices regarding treatment plans.

In the Supplemental materials, Table 1 showcases studies effectively using the SVM algorithm for cancer classification tasks, highlighting its practical impact and value.

### Decision Tree and Random Forest

**Figure 6.**
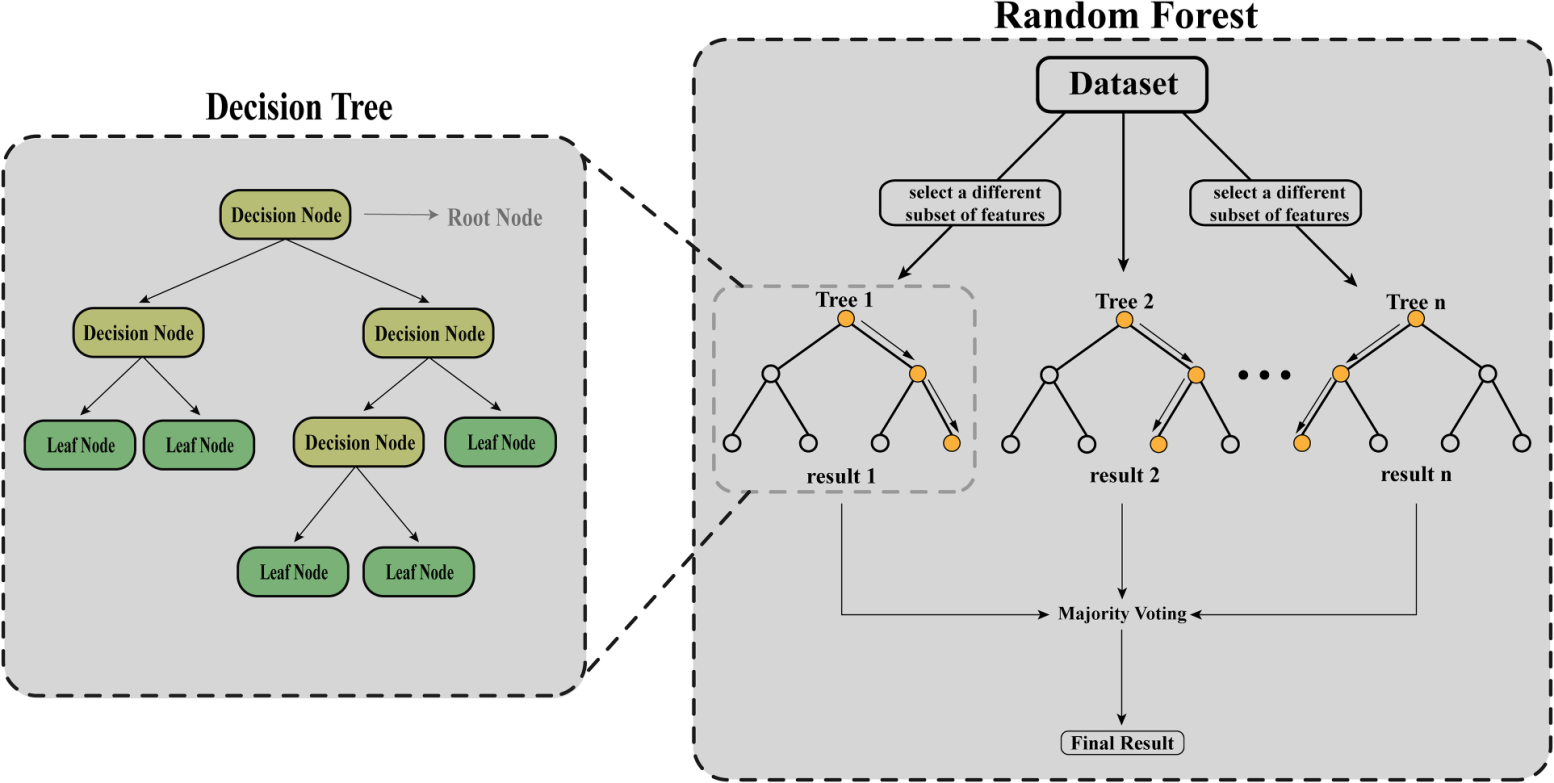
Structure of the decision tree and random forest algorithms.

Decision trees and random forests serve as machine-learning algorithms that derive conclusions through a series of inquiries, similar to a flowchart. As depicted in Figure 6, decision trees engage a series of binary questions regarding the dataset, branching at each stage (decision nodes) to arrive at a conclusive decision/final result based on majority voting. In contrast, random forests comprise several decision trees, which collectively operate on distinct feature subsets to yield a more precise and consistent prediction. For instance, in cancer research, these models can be employed to categorize tumors as benign or malignant. A decision tree might pose queries such as “Does the tumor exceed 2 cm in size?” or “Do the cells exhibit irregular shapes?” The tree navigates the decision-making pathway based on the responses until a final categorization is determined. In the case of a random forest model, multiple decision trees are utilized, each examining a random data subset, and their outcomes are combined via voting to produce a more reliable classification.

In the Supplemental materials, Table 2 details significant studies that have employed decision trees and random forests algorithms in cancer research, illustrating their practical uses and contributions to classification challenges.

### K-Nearest Neighbors

**Figure 7.**
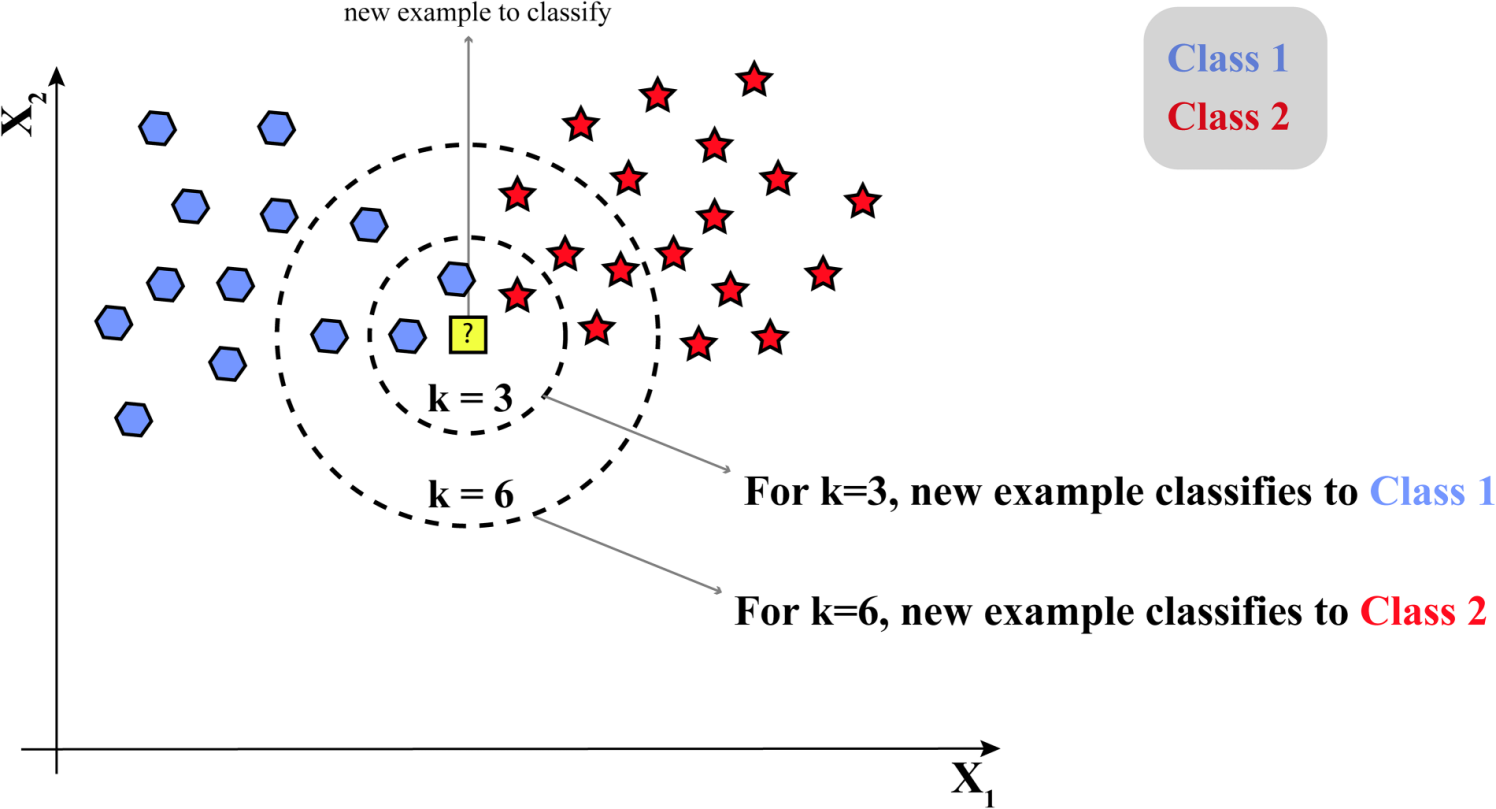
Structure of the K-nearest neighbors (KNN) algorithm.

The KNN model is a simple and intuitive machine-learning algorithm that classifies data based on the properties of its closest neighbors. As shown in Figure 7, KNN identifies the K most similar data points to a new, unknown data point and assigns it to the majority class of its neighbors. For instance, in cancer research, KNN can be used for various purposes, such as predicting the response to a specific treatment or determining cancer subtypes. If a new patient's tumor characteristics are input into the KNN model, it will identify the K most similar cases from the existing data. Then, it will determine the majority outcome among those neighbors, such as a successful treatment response or a specific cancer subtype, and predict that the new patient will likely experience a similar outcome.

In the Supplemental materials, Table 3 compiles studies that have leveraged the KNN algorithm in cancer research, emphasizing its adaptability and practical applications in classification tasks.

### K-Means

**Figure 8.**
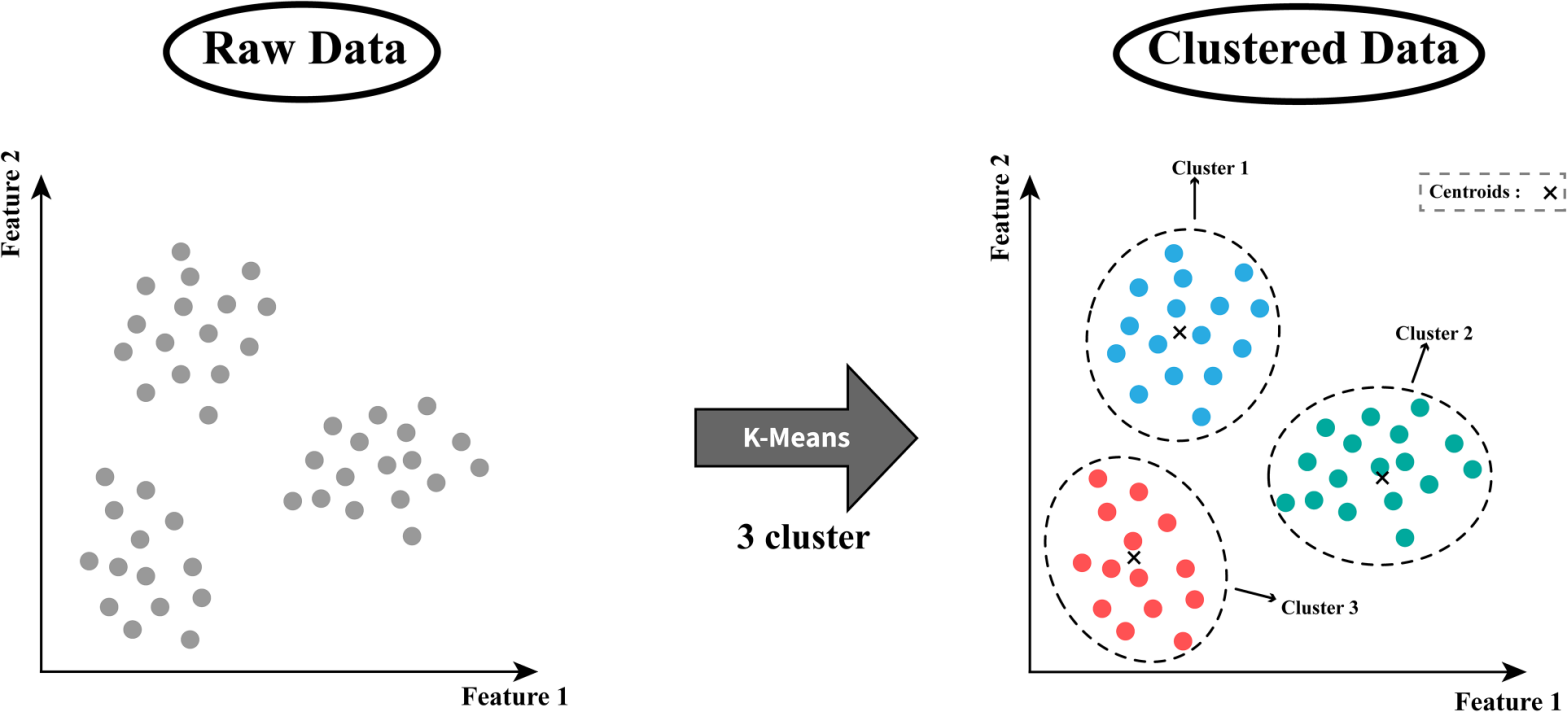
Structure of the K-means algorithm.

The k-means model is a straightforward clustering algorithm that groups data points based on their similarity. This algorithm is illustrated in Figure 8 and works by identifying the “k” number of cluster centroids (centers) and assigning each data point to the nearest centroid, creating distinct clusters. In cancer research, k-means is applied for clustering purposes, such as discovering subgroups of patients with similar gene expression patterns or tumor characteristics. For example, given a gene expression data dataset from different patients, the k-means algorithm would find “k” centroids representing distinct gene expression profiles. Then, each tumor is assigned to the nearest centroid, creating groups of tumors with similar gene expression patterns. This information can help researchers identify new cancer subtypes or understand the underlying biological mechanisms.

In the Supplemental materials, Table 4 gathers key studies employing the k-means algorithm in cancer research, demonstrating its practical uses and contributions to solving clustering issues.

### Logistic Regression

**Figure 9.**
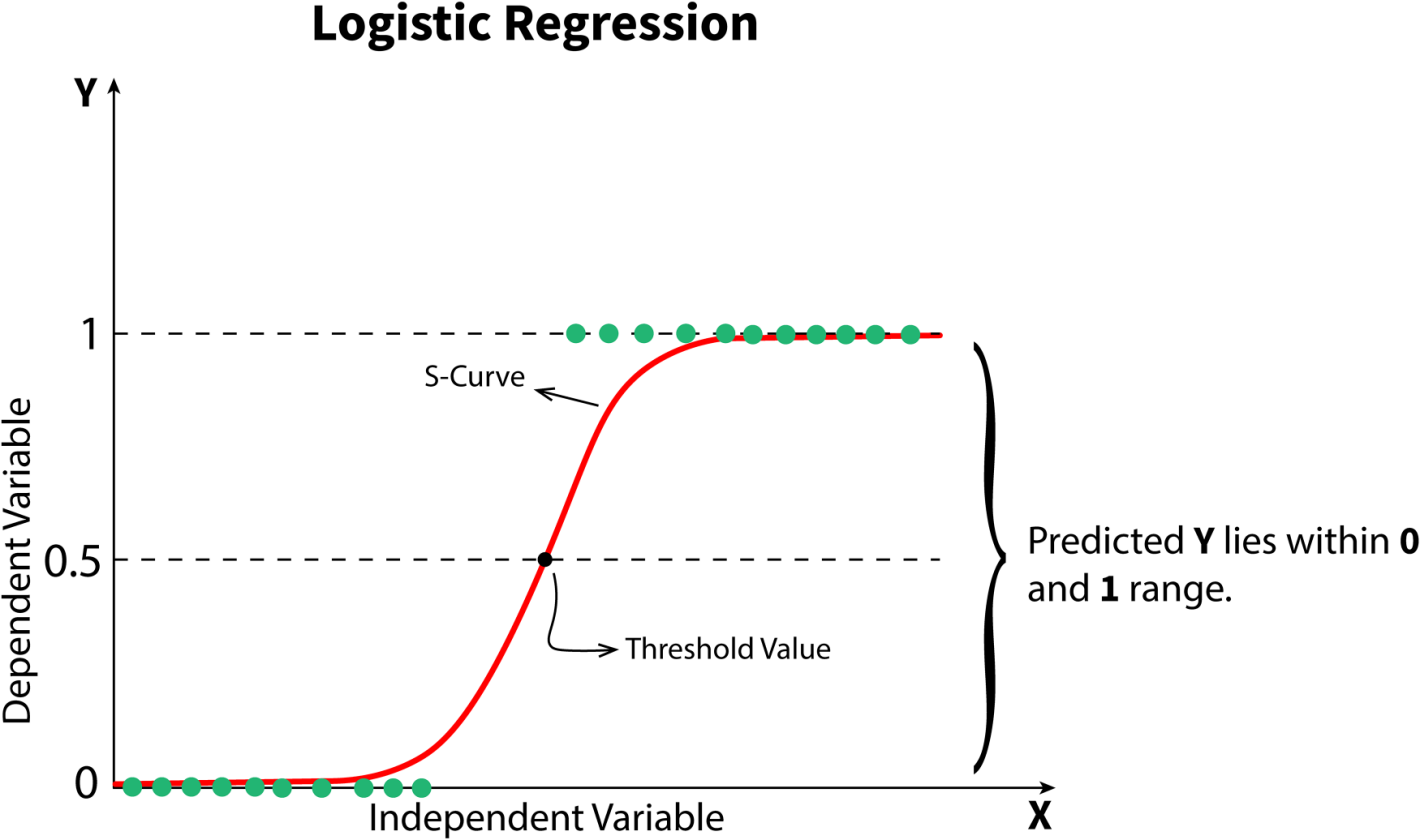
Structure of the logistic regression algorithm.

Logistic regression is a model that predicts the probability of an event occurring on the basis of one or more input variables. As shown in Figure 9, logistic regression uses a particular S-shaped curve, called the logistic function, to model the relationship between the variables. This allows it to produce probabilities between 0 and 1, which can be converted into binary outcomes using a threshold value. In cancer research, logistic regression is applied for various purposes, such as predicting the likelihood of a patient's tumor being malignant based on factors such as age, tumor size, and genetic markers. The model generates an S-shaped curve representing the probability of malignancy as a function of the input variables. Researchers can classify tumors as malignant or benign by setting a threshold on this curve.

In the Supplemental materials, Table 5 outlines important studies that have used the logistic regression model in cancer research, underscoring its practical implementations and importance in addressing classification challenges.

### Naïve Bayes

**Figure 10.**
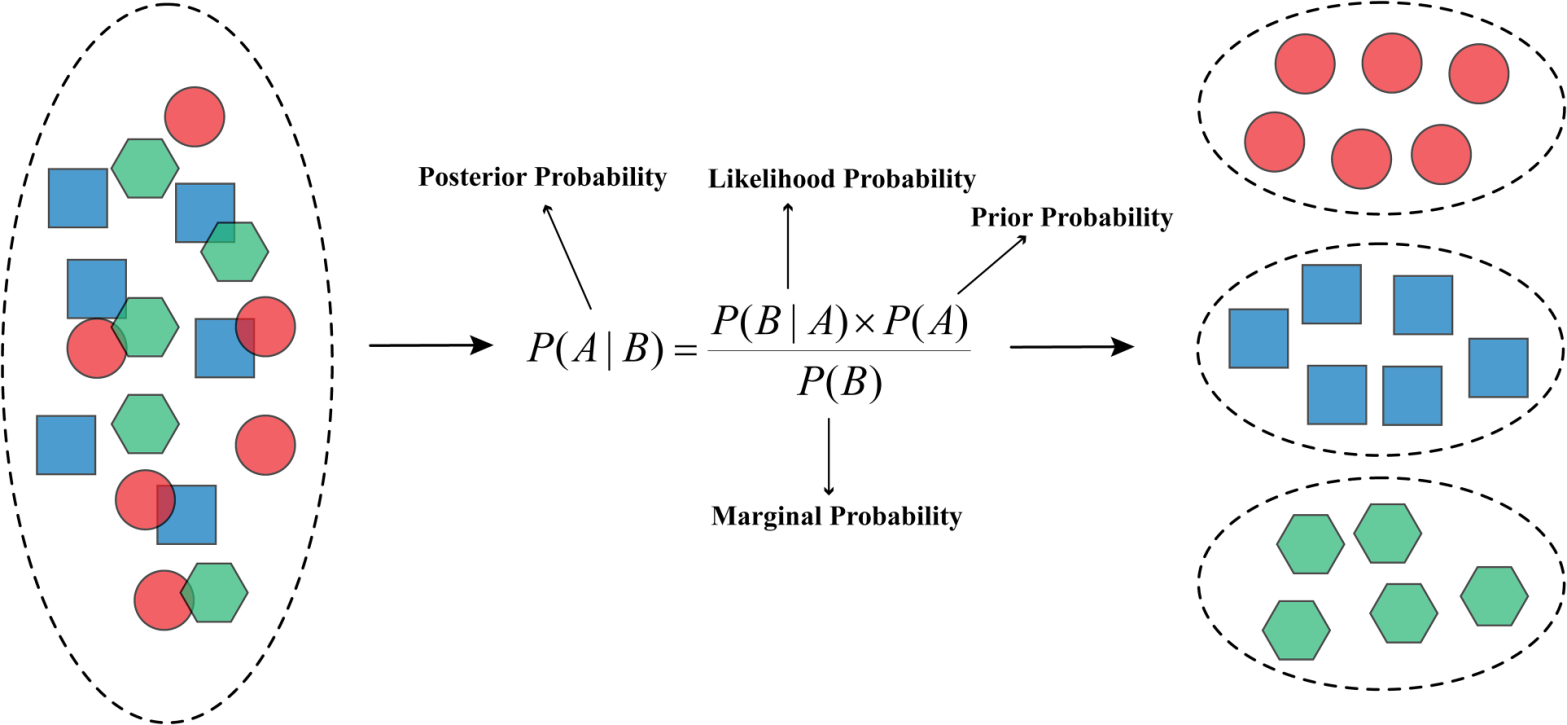
Structure of the Naïve Bayes algorithm.

The Naïve Bayes model is a simple yet powerful probabilistic classifier based on Bayes’ theorem. It works by calculating the probability of each class (eg, benign or malignant tumor) given the input features, assuming that the features are conditionally independent. As shown in Figure 10, the model combines prior probabilities (known information about the classes), likelihoods (the probabilities of observing the features given a class), and marginal probabilities (the probabilities of observing the features) to compute the posterior probabilities, which represent the updated probabilities of each class. In cancer research, the Naïve Bayes model can be used for classification, such as diagnosing cancer based on a set of symptoms or medical test results. Given the input features, the algorithm calculates the posterior probabilities of each class (eg, cancerous or non-cancerous) and assigns the unknown case to the class with the highest probability.

In the Supplemental materials, Table 6 presents studies that have applied the Naive Bayes algorithm in cancer research, showing its practical uses and effects on classification issues.

### eXtreme Gradient Boost

**Figure 11.**
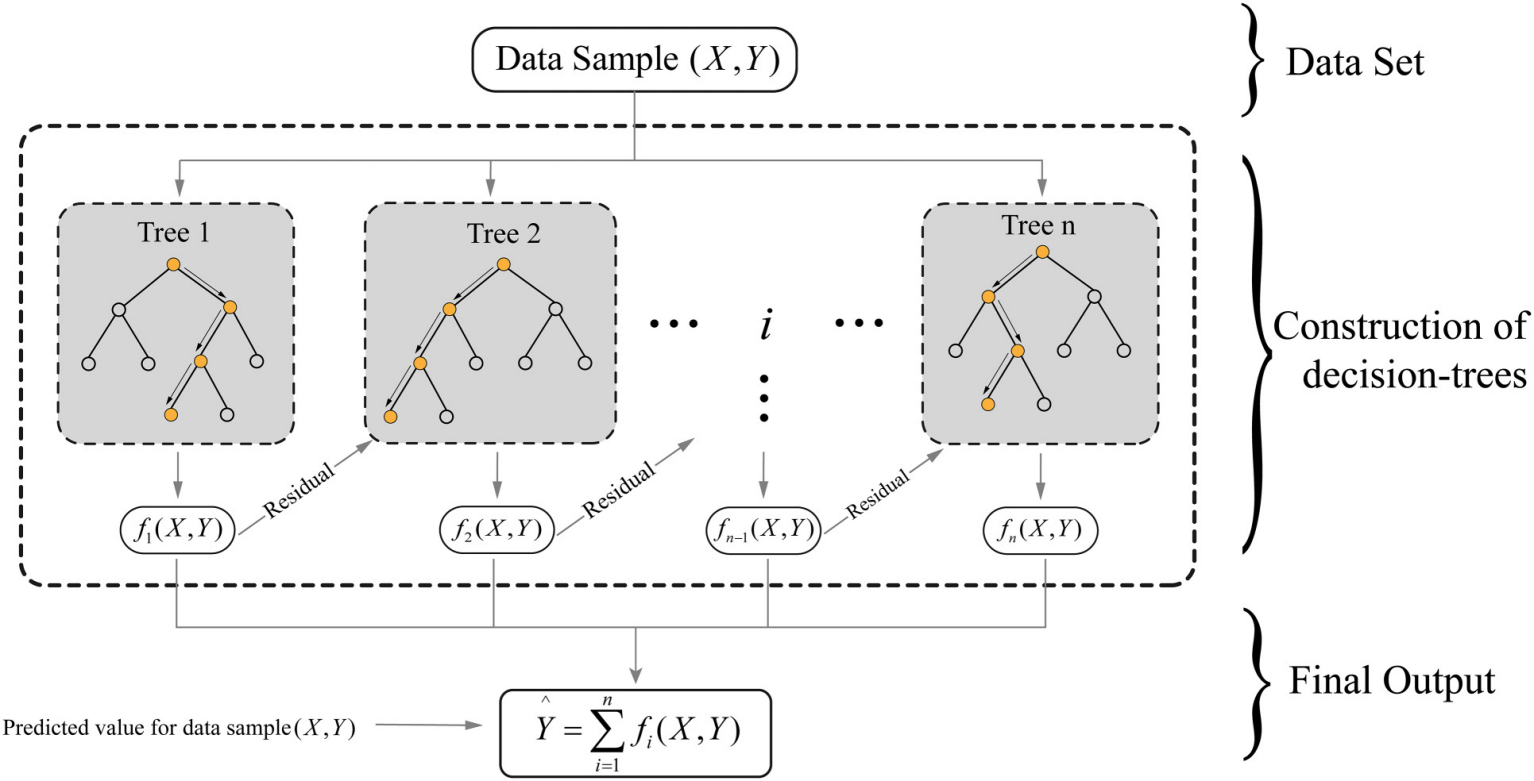
Structure of the eXtreme Gradient Boosting (XGBoost) algorithm.

The XGBoost model is an advanced machine learning algorithm that builds on the concept of boosting, which combines multiple weak models to create a robust and accurate model. As shown in Figure 11, in XGBoost, a series of decision trees are constructed sequentially, with each tree focusing on correcting the errors (residual values) made by the previous tree in the series. This process continues until a specified stopping criterion is met, resulting in a powerful classifier. In cancer research, the XGBoost model can be employed for classification purposes, such as predicting cancer recurrence based on patient characteristics, genetic data, and treatment history. By iteratively building decision trees that focus on correcting previous errors, XGBoost generates a model that can accurately distinguish between patients with high and low risks of cancer recurrence.

In the Supplemental materials, Table 7 compiles significant studies using the XGBoost algorithm in cancer research, emphasizing its practical applications and efficacy in classification tasks.

### Hybrid Models

Hybrid machine learning algorithms refer to the combination of different machine learning methods or models to create intelligent algorithms that leverage the strengths of each individual component. These algorithms improve overall performance, enhance accuracy, or address specific challenges that cannot be effectively tackled by a single method alone.

**Figure 12.**
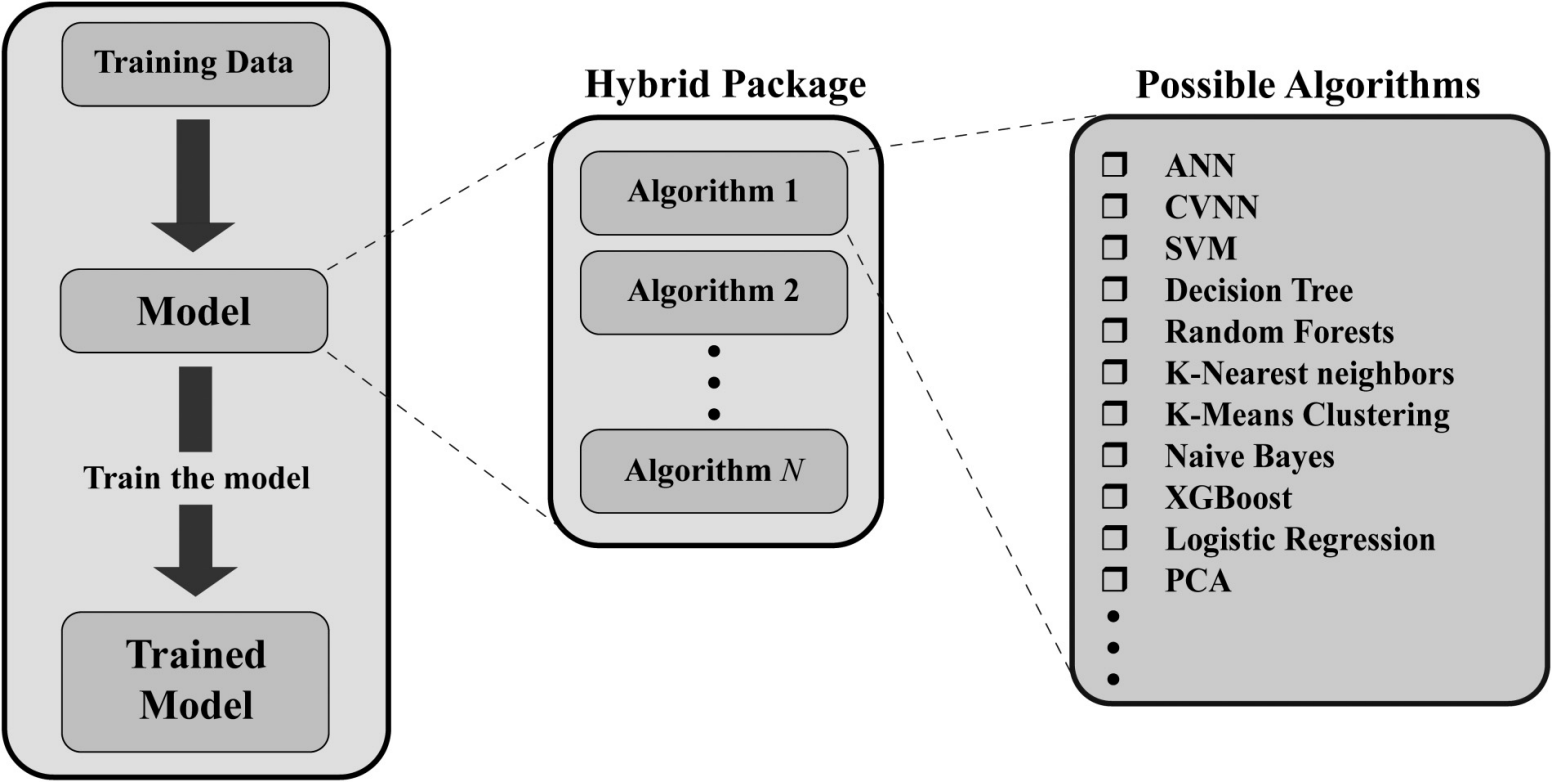
The structure of the hybrid algorithm (ANN: artificial neural networks, CVNN: complex-valued neural network, SVM: support vector machine, XGBoost: eXtreme Gradient Boost, PCA: principal component analysis).

For example, a hybrid machine-learning algorithm could combine the power of decision trees, SVM, and ANNs to classify tumor types based on genetic markers, patient characteristics, and medical imaging data. Decision trees can capture simple decision rules, SVMs can handle complex decision boundaries, and ANNs can learn intricate patterns from the data. By integrating these techniques (Figure 12), the hybrid algorithm can leverage the complementary strengths of each method to achieve more accurate and robust tumor classification.

This hybrid approach allows for a more comprehensive analysis of cancer data, taking advantage of multiple machine-learning techniques to tackle the complexity and heterogeneity of cancer-related datasets. Combining different methods intelligently, hybrid algorithms can improve classification and prediction outcomes, leading to better insights and decision-making in cancer research.

In the Supplemental materials, Table 8 features studies that have effectively used hybrid algorithms in cancer research for classification tasks, showcasing their real-world effectiveness and value.

**Figure 13.**
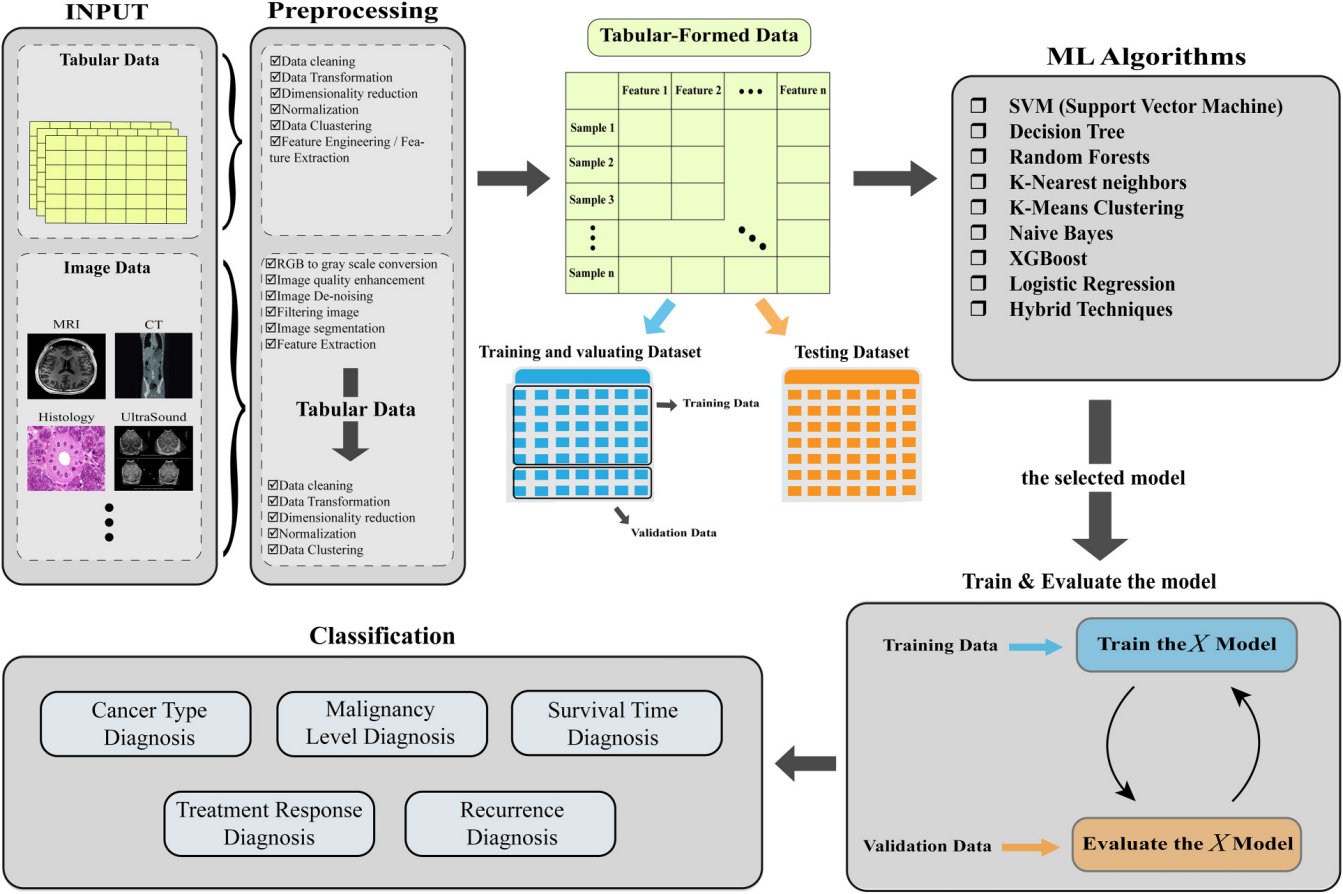
A brief overview of the necessary steps for applying different ML algorithms to cancer research.

Figure 13 summarizes the steps for applying different types of machine learning algorithms to cancer research, which have already been described one by one.

## Soft Computing Application in Cancer Research

Soft computing algorithms, which include fuzzy logic and evolutionary algorithms optimized using evolutionary algorithms, have shown great promise in advancing cancer research. Fuzzy logic provides a means of handling uncertainty and imprecision in medical data, allowing for more accurate diagnoses and personalized treatment plans. Conversely, ANNs can learn complex relationships between input data and output variables, making them helpful in predicting cancer outcomes and identifying novel biomarkers. Evolutionary optimization algorithms, such as genetic algorithms and particle swarm optimization, can efficiently search through large datasets to find optimal solutions, thus improving cancer diagnosis and treatment planning accuracy. By incorporating these soft computing techniques into cancer research, researchers have significantly improved patient outcomes, including earlier detection and more effective treatment options. In the following section, we describe each algorithm and then include a separate table covering the most significant works using each algorithm.

### Fuzzy Inference System

**Figure 14.**
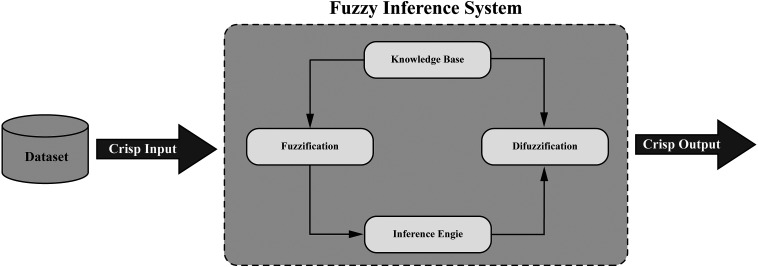
Structure of the fuzzy inference system (FIS) algorithm.

The fuzzy inference system (FIS) is a computational framework that deals with uncertainty and imprecision using fuzzy logic, which allows for partial truth values between 0 and 1 rather than binary true or false. As shown in Figure 14, the FIS consists of 4 main steps: “Fuzzification,” “Knowledge Base,” “Inference Engine,” and “Defuzzification.” In cancer research, FIS is used for classification and prediction purposes, such as determining the risk of cancer recurrence or the effectiveness of a specific treatment. For example, input variables such as tumor size, age, and genetic markers may have associated uncertainty. FIS enables the processing of this uncertain information through fuzzification, which converts the crisp input values into fuzzy sets representing degrees of membership in different categories. The fuzzy inference engine uses a knowledge base to apply rules to the fuzzy input data to generate fuzzy output values. Finally, defuzzification converts the fuzzy output values back into crisp values, providing a precise prediction or classification, such as the low or high risk of cancer recurrence.

In the Supplemental materials, Table 9 outlines prominent studies utilizing the Fuzzy Inference System in cancer research.

### Hybrid Soft Computing Algorithms

**Figure 15.**
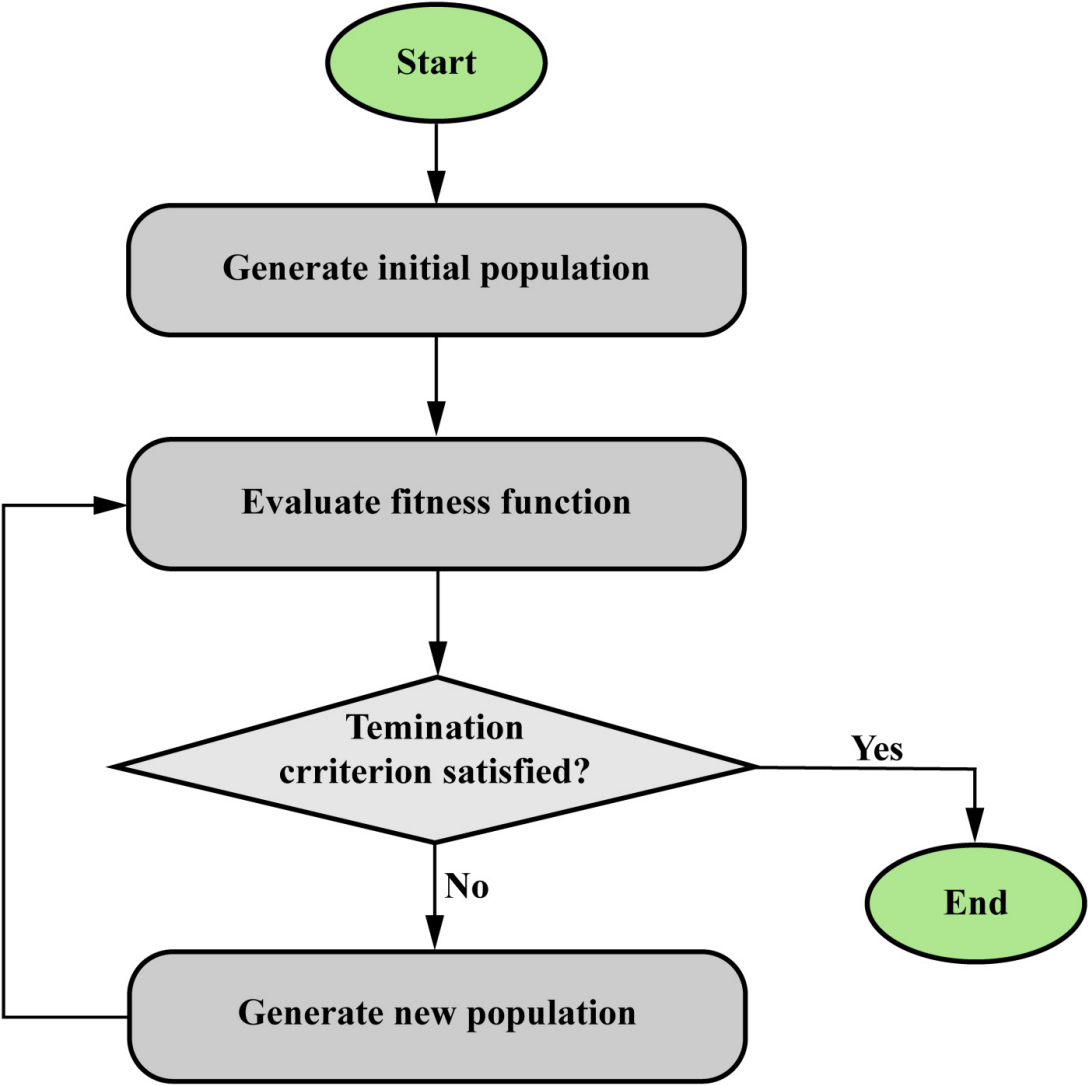
Structure of optimization algorithms (evolutionary algorithms).

Hybrid soft computing algorithms that combine optimization methods and ANN or FIS offer a powerful approach to solving complex problems in cancer research. As shown in Figure 15, optimization methods, such as genetic algorithms or particle swarm optimization, are used to fine-tune the ANN or fuzzy systems, thereby enhancing their performance and adaptability. In cancer research, a hybrid algorithm that combines optimization methods and ANN or FIS can be applied for classification and prediction purposes. For example, imagine a scenario in which researchers want to predict the effectiveness of a specific cancer treatment based on a patient's genetic data and clinical variables. First, the FIS is designed to process and analyze the input data using fuzzy rules that account for the inherent uncertainty and imprecision in the information. The FIS outputs a prediction of treatment effectiveness in the form of a fuzzy membership value. Next, an optimization method, such as a genetic algorithm, is employed to fine-tune the FIS parameters, including the fuzzy rules, membership functions, and aggregation operators. This optimization process iteratively refines the fuzzy system's performance, resulting in a more accurate and reliable prediction model.

By combining the strengths of FIS and optimization methods, hybrid soft computing algorithms offer a robust and flexible approach to cancer classification and prediction tasks, capable of handling the complex and uncertain nature of cancer-related data.

In the Supplemental materials, Table 10 compiles important studies that have used optimization algorithms with ANN or FIS in cancer research, emphasizing their practical uses and effectiveness in the field.

## Deep Learning

Deep learning algorithms are advanced computational models capable of processing and analyzing complex visual data. These algorithms include ANNs, CNN, multimodal CNN, and recurrent neural networks (RNNs) combined with long short-term memory (LSTM) cells. These models are designed to address specific challenges in processing and learning visual information.

**Figure 16.**
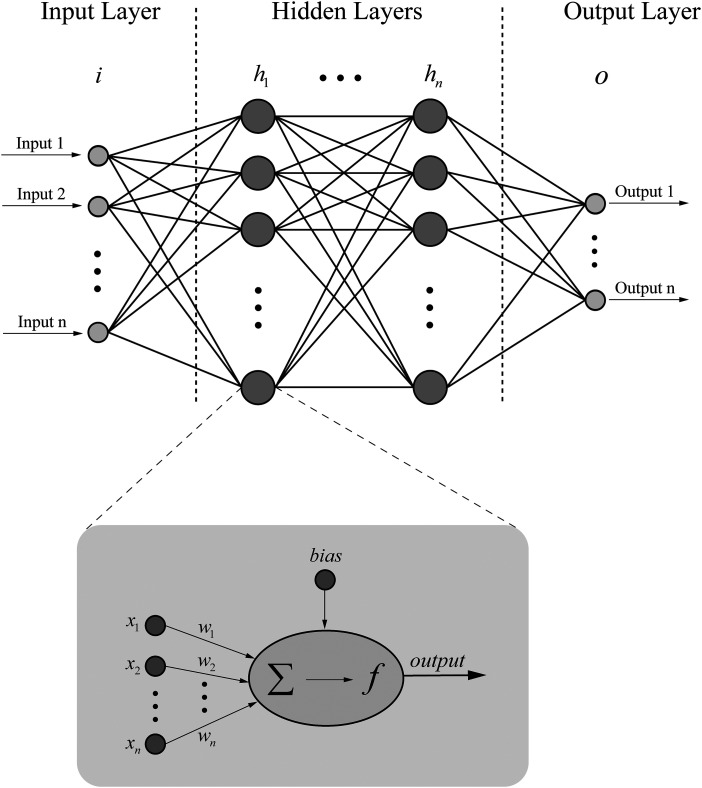
Structure of the artificial neural network (ANN).

ANNs are computational models inspired by the structure and functioning of the human brain. As shown in Figure 16, in an ANN applied to cancer research, the inputs consist of relevant data features such as patient demographics, tumor characteristics, genetic markers, and medical images. These input features are passed through multiple layers of neurons, where they are processed and transformed. Each layer consists of numerous interconnected neurons that apply weights and biases to the incoming data, enabling the network to learn complex relationships and patterns. After the information passes through the hidden layers, it reaches the output layer, which generates the final predictions or classifications, such as the likelihood of cancer recurrence or the type of cancer. The output layer is designed to match the desired format, such as a single output for binary classification or multiple outputs for multi-class classification. In summary, ANN processes cancer-related input data through multiple interconnected layers with adjustable weights and biases and generates outputs relevant to cancer classification or prediction tasks.

**Figure 17.**
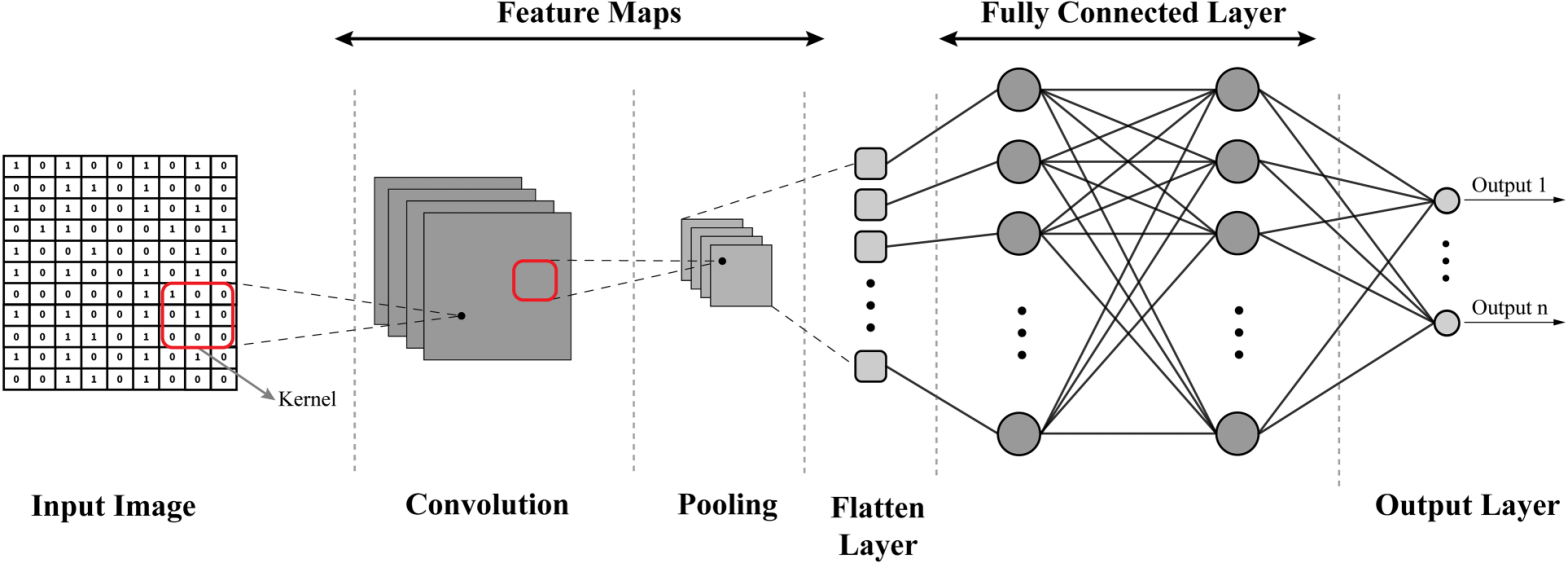
Structure of convolutional neural network (CNN).

CNNs (depicted in Figure 17) are specialized deep-learning models designed to process grid-like data, such as images. They use different layers, such as convolutional, pooling, and flattened layers, to scan and identify local patterns or features in an image, such as edges or textures. In cancer research, CNNs can be used for tasks such as classifying histopathological images, identifying cancerous cells, or detecting tumors in medical imaging data, such as MRI or CT scans.

**Figure 18.**
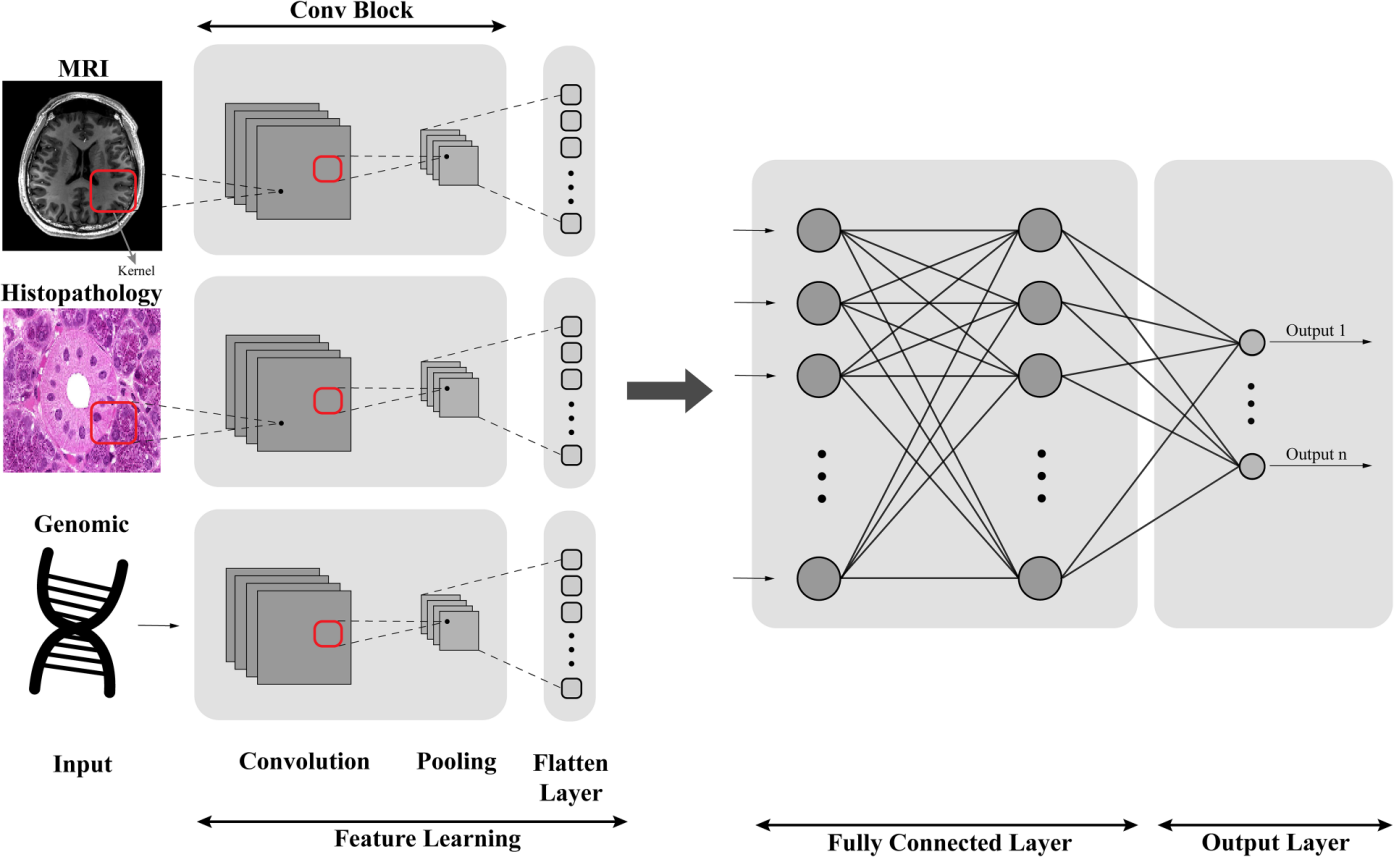
Structure of a multimodal CNN.

Multimodal CNNs are an extension of traditional CNNs and are designed to process and integrate multiple data types, such as images, text, and audio. As illustrated in Figure 18 by combining information from different sources, these networks can improve classification or prediction performance. In cancer research, a multimodal CNN could be used to simultaneously predict data (eg, patient demographics, and medical history), leading to more accurate and comprehensive predictions.

**Figure 19.**
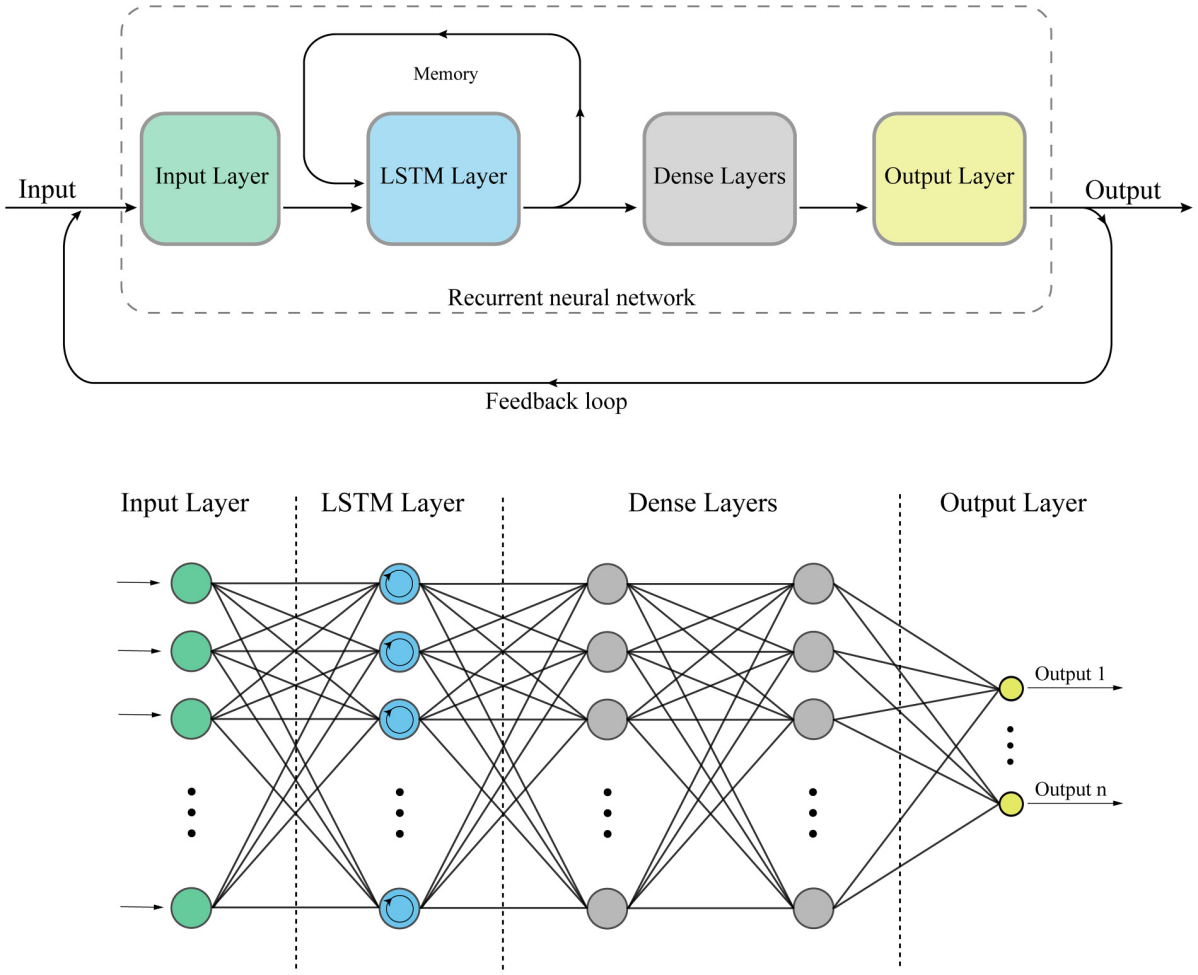
Structure of a recurrent neural network (RNN) and long short-term memory (LSTM) cell.

Figure 19 depicts the structures of RNNs and LSTM. RNNs are deep learning models designed to process sequential data, making them suitable for tasks involving time series or ordered data. LSTM cells are a type of RNN architecture that addresses the challenge of learning long-term dependencies in the data. In cancer research, RNNs with LSTM cells can be applied to tasks such as predicting cancer progression or recurrence based on time-series data, such as gene expression levels or patient vital signs collected over time.

In summary, deep learning algorithms, including ANNs, CNNs, multimodal CNNs, and RNNs with LSTM cells, offer powerful tools for processing complex visual data in cancer research, enabling accurate classification and prediction tasks that can improve patient outcomes and advance our understanding of cancer biology.

In the Supplemental materials, Table 11 details significant studies using deep learning algorithms in cancer research, demonstrating their practical applications and influence in the field.

## Conclusions and Future Work

Rapid progress in AI has profoundly impacted cancer research and treatment, leading to enhanced patient outcomes and healthcare efficiency. Incorporating AI algorithms in cancer diagnosis, prognosis, and treatment response prediction has facilitated early detection, customized intervention approaches, and improved overall patient care. In this comprehensive guide, we have explored the crucial role of AI in cancer research, with a specific focus on the applications of machine learning, soft computing, and deep learning algorithms. We have offered an in-depth overview of various algorithms’ functionality and particular applications, supported by pertinent figures and a tabular summary of key findings from each study with the lowest complexity and high suitability for a better understanding of all readers with different backgrounds.

The impressive advantages of AI-driven algorithms in cancer care emphasize their potential to reshape cancer research and clinical practice. This review serves as an invaluable resource for researchers, clinicians, and healthcare industry stakeholders, offering insights into AI's present state and future potential in cancer care.

Future research in AI for cancer care could explore:

1. Developing advanced AI algorithms to enhance the precision and efficiency of cancer care.
2. Utilizing multi-modal data (eg, genomic, proteomic, imaging, and clinical reports) to gain a comprehensive understanding of cancer, focusing on AI's ability to process and analyze such diverse information.
3. Creating personalized treatment plans using AI to consider individual patient characteristics, aiming for treatments that are both effective and have minimal side effects.
4. Leveraging AI in drug discovery to quicken the identification of drug targets and optimize drug designs, potentially speeding up the creation of new cancer treatments.
5. Addressing ethical and regulatory challenges associated with AI in cancer care, such as data privacy and algorithmic fairness, to ensure AI's responsible use.

In conclusion, the future of AI in cancer research and treatment holds tremendous promise for enhancing patient outcomes and healthcare efficiency. Ongoing innovation and collaboration among researchers, clinicians, and industry partners will be vital for unlocking the full potential of AI in revolutionizing cancer care.

## Supplemental Material

sj-docx-1-tct-10.1177_15330338241250324 - Supplemental material for The Application of Artificial Intelligence to Cancer Research: A Comprehensive Guide Supplemental material, sj-docx-1-tct-10.1177_15330338241250324 for The Application of Artificial Intelligence to Cancer Research: A Comprehensive Guide by Amin Zadeh Shirazi, Morteza Tofighi, Alireza Gharavi and Guillermo A. Gomez in Technology in Cancer Research & Treatment

## Abbreviations

AI: artificial Intelligence
KNN: K-nearest neighbors; ANN artificial neural network
CNN: convolutional neural network
RNN: recurrent neural network
micro-FTIR: micro-Fourier transform infrared spectroscopy
ABC: artificial bee colony
RC: random committee
MRMR: minimum redundancy maximum relevance
TLBO: teaching learning-based optimization
FCM: fuzzy cognitive maps
WDBC: Wisconsin diagnostic breast cancer
WBC: Wisconsin breast cancer
MRI: magnetic resonance imaging
TCGA: Cancer Genome Atlas
LIDC: Lung Image Database Consortium
ROI: region of interest
mpMRI: multiparametric magnetic resonance imaging
SELDI: surface-enhanced laser desorption and ionization
CEA: carcinoembryonic antigen
GLCM: gray level co-occurrence matrix
WRBC: Wisconsin Recurrence Breast Cancer Database
ABCD: Asymmetry Index (A), Border Irregularity (B), Color score (C) and Diameter (D)
MGI: maximum gradient intensity
FNA: fine needle aspiration
DSS: disease-specific survival
NYU: New York University
NN-ET: neural network and extra tree
UCSB: University of California, Santa Barbara, USA
RBCC: red blood cell count
TS: tumor size
HBP: high blood pressure
CRP: C-reactive protein
FSES: fuzzy soft expert system
MIAS: Mammographic Image Analysis Society
T2FIS: type II fuzzy inference system
PCA: principal component analysis
SOM: self-organizing map
BI-RADS: breast imaging reporting and data system
IWOA: improved whale optimization algorithm
PSO: particle swarm optimization
EO: equilibrium optimizer
DNN: deep neural network
WBC: white blood cells
PT: prothrombin time
TT: thrombin time
INR: international normalized ratio
RBC: red blood cells
HMLP: hybrid multi-layered perceptron
TCM: traditional Chinese medicine
BOAALO: butterfly optimization algorithm and the ant lion optimizer
MLP: multilayer perceptron
BILSTM: bidirectional long short-term memories
CSE: concurrent squeeze and excitation
PSA: prostate-specific antigen
BPH: benign prostatic hyperplasia
FNB: fine needle biopsy
ELM: extreme learning machine
TAI-net: triple-attention interaction network
DWI: diffusion-weighted Imaging
XGboost: eXtreme gradient boost
ANFIS: adaptive network-based fuzzy inference system
LSTM: long-term short-term memory networks
ML: machine learning
CS: Cuckoo search
CFS: correlation feature selection
SBO: satin bowerbird optimization
IWO: invasive weed optimization
QDA: quadratic discriminant analysis
T1F: type-1 fuzzy logic
WPBC: wisconsin prognostic breast cancer
CT Scan: computed tomography scan
GEO: gene expression omnibus
BreakHis: Breast Cancer Histopathology Database
MCC: Matthew's correlation coefficient
AUC: area under the curve
SEER: surveillance, epidemiology, and end results
CRlncRC: cancer-related long non-coding RNAs
GSI: gemstone spectral imaging
BMI: body mass index
ISIC: international skin imaging collaboration
LBP: local binary patterns
SF: short form
OS: overall survival
CVC: central venous catheter
PLS-ANNDA: partial least squares artificial neural network discriminant analysis
miRNA: micro-ribonucleic acids
FIS: fuzzy inference system
FP: flank pain
VHLG: Von Hippel–Lindau Gene
TCEE: trichloroethylene exposure
AJCC: American Joint Committee on Cancer
DDSM: Digital Database for Screening Mammography
BRATS: multimodal brain tumor image segmentation benchmark
EM: expectation maximization
CART: classification and regression trees
CVNN: complex-valued neural network
ODNN: optimal deep neural network
GONN: genetically optimized neural network
ASO: atom search optimization
GA: genetic algorithm
TNM: Tumor (T), Node (N), and Metastasis (M)
FIB: fibrinogen
APTT: activated partial thromboplastin time
ALB: albumin
PLT: platelet count
GLB: globulin
ELM: extreme learning machine
MULTP: modified uniform local ternary patterns
Lung-PET-CT-Dx: large-scale CT and PET/CT dataset for lung cancer diagnosis
SERS: surface-enhanced Raman spectroscopy
PCa: prostate cancer
HUAP: University Hospital Antônio Pedro
TL: transfer learning
EMS-Net: ensemble of multiscale convolutional neural networks
ADC: obvious dispersion coefficient
BEiT: BERT pre-training of image transformers.
