# Supplementary material for: The Application of Artificial Intelligence to Cancer Research: A Comprehensive Guide
Source: Technol Cancer Res Treat. 2024 May 22;23:15330338241250324. doi: 10.1177/15330338241250324 (PMC11113055; doi:10.1177/15330338241250324)
Supplement: sj-docx-1-tct-10.1177_15330338241250324 - Supplemental material for The Application of Artificial Intelligence to Cancer Research: A Comprehensive Guide [file sj-docx-1-tct-10.1177_15330338241250324.docx]

| **Table 1. Published works that have implemented the support vector machine (SVM) model in cancer research.** | | | | | | | | | | | | |
| --- | --- | --- | --- | --- | --- | --- | --- | --- | --- | --- | --- | --- |
| Ref | Year | | Dataset | Cancer Type | Number of Patients | | Method Name | Input | Data Preprocessing Technique | Clinical Endpoint | Model Validation Technique | Results |
| [69] | | 2009 | mini-MIAS database ([Link](http://peipa.essex.ac.uk/info/mias.html)) | Breast | | 75 | SVM | Mammographic images | 1. Image Enhancement  2. Thresholding Segmentation  3. Feature Extraction | Tumour features classification | NA | * Sensitivity: 88.75% |
| [70] | | 2019 | MIAS database ([Link](https://www.repository.cam.ac.uk/handle/1810/250394)) | Breast | | 95 | SVM | Mammographic images | 1. image enhancement  2. segmentation using the maximization method (to remove pectoral muscle and retain the breast region)  3. feature extraction using the Hough transform | Malignancy level (malignant – benign) | NA | Accuracy:  94% |
| [71] | | 2009 | 1. WDBC ([Link](https://archive.ics.uci.edu/ml/datasets/breast+cancer+wisconsin+(diagnostic)))  2. WPBC ([Link](https://archive.ics.uci.edu/ml/datasets/breast+cancer+wisconsin+(Prognostic))) | Breast | | WDBC: 569  WPBC:  198 | SVM | For each cell nucleus:  1. radius  2. texture  3. perimeter,  4. area,  5. smoothness  6. compactness  7. concavity  8. Concave points  9. symmetry,  10. fractal dimension  11. Tumor size (WPBC)  12. Lymph node status (WPBC) | NA | 1) malignancy level (malignant – benign)  2) recurrence (C1: 1 to 12 months – C2: 1 to 3 years – C3: 3 to 6 years – C4: more than 6 years) | 10-fold cross-validation | Accuracy > 97%  Sensitivity > 96%  Specificity > 98%  2) Accuracy: 95.61%  Sensitivity: 95.97%  Specificity: 95.57% |
| [72] | | 2016 | NA | Lung | | 6 CT scan images, 15 MRI images | SVM | CT scan, MRI, and ultrasound images | 1. image enhancement  2. De-noising image  3. image segmentation using superpixel segmentation (separation of interest areas from other areas of the picture)  4. feature extraction (such as area of interest, shape, size of the nodule, and contrast enhancement) | Malignancy level (malignant – benign) | NA | * Accuracy: 89.5% |
| [73] | | 2017 | Gene Expression Omnibus (GEO)  [Link](http://www.ncbi.nlm.nih.gov/geo/): GSE17537, GSE38832, GSE28812  2) Cancer Genome Atlas (TCGA; [Link](http://tcga-data.nci.nih.gov/tcga/)) | colon | | 708 | SVM | Expression level of genes, age, sex, stage, death status, follow-up time, survival time | 1) Data normalization  2) Data transformation | Recurrence susceptibility (recurrence – no recurrence) | 5-fold cross-validation | Accuracy: 92% |
| [74] | | 2017 | UCI-WBC dataset ([Link](https://archive-beta.ics.uci.edu/dataset/15/breast+cancer+wisconsin+original)) | Breast | | 699 | Two-Step-SVM | 1) Radius [mean of distances from the center to points on the perimeter]  2) Texture [standard deviation of grey-scale values]  3) Perimeter  4) Area  5) Smoothness  6) Compactness  7) Concavity ( severity of concave portions of thecontour]  8) Concave points  9) Symmetry  10) Fractal dimension | Data clustering using a two-step algorithm | Malignancy level (malignant – benign) | 10-fold cross-validation | Accuracy: 99.1% |
| [75] | | 2020 | NA | Bone | | 200 | SVM | MRI images | 1) Filter the image  2) Image segmentation (better expression of the area of interest)  3) Statistical feature extraction | Diagnosis (Cancer cell detection: cancerous or non-cancerous) | NA | Accuracy: 92%  Specificity: 91%  Sensitivity: 93% |
| [76] | | 2017 | LIDC ([Link](https://wiki.cancerimagingarchive.net/pages/viewpage.action?pageId=1966254)) | Lung | | NA | SVM | CT scan images | 1) preprocessing (such as de-noising)  2) segmentation (extracting Region of Interest)  3) feature extraction | Malignancy level (malignant – benign) | NA | Accuracy: 95.16%  Sensitivity: 98.21%  Specificity: 78.69% |
| [77] | | 2020 | BreakHis ([Link](https://web.inf.ufpr.br/vri/databases/breast-cancer-histopathological-database-breakhis/)) | Breast | | 82 | Cubic SVM | Histopathologic images | 1) Preprocessing (RGB to grayscale conversion)  2) feature extraction (Texture Feature: First Order Statistics–Graph Features–Morphological Features: Radius, area, perimeter, size, shape,  Roundness, smoothness) | Malignancy level (malignant – benign) | 10-fold cross-validation | Accuracy: 88.91%  Sensitivity: 81.85%  Specificity: 82.22%  F1-score: 82.48% |
| [78] | | 2016 | mini-MIAS database | Breast | | 279 (192 train examples–87 test examples) | SVM | Mammographic images | 1) preprocessing (such as de-noising)  2) segmentation and thresholding  3) feature extraction | Malignancy level (malignant – benign) | NA | Accuracy: 96.55%  Sensitivity: 96.97%  Specificity: 96.29% |
| [79] | | 2019 | NA | prostate | | 400 | SVM | Histopathologic images | 1) ROI segmentation  2) feature extraction (morphological features of the cell nucleus and lumen: area, perimeter, major axis length, minor axis length, circularity, diameter, nucleus-to-nucleus distance, nucleus-to-nucleus minimum distance, eccentricity, compactness) | Malignancy level (benign – malignancy grade 3–malignance grade 4–malignance grade 5) | 2-fold cross-validation | Accuracy: 88.73%  Sensitivity: 89.43%  Specificity: 89.93%  MCC: 75.2% |
| [80] | | 2018 | NA | prostate | | 48 | SVM | mp-MRI pictures | 1) Data Cleaning  2) Data transformation | Malignancy level (malignant – benign) | 10-fold cross-validation | Accuracy:  87%  Sensitivity: 91.2%  Specificity: 89.05%  AUC: 95% |
| * Some studies included in our review did not provide a gold standard, such as the Accuracy metric, for evaluation. As a result, the assessment of these studies may present some challenges due to the absence of a standardized benchmark. (SVM: Support Vector Machine, MIAS: Mammographic Image Analysis Society, WDBC: Wisconsin Diagnostic Breast Cancer, WPBC: Wisconsin Prognostic Breast Cancer, CTScan: Computed Tomography Scan, MRI: Magnetic Resonance Imaging, TCGA: The Cancer Genome Atlas, LIDC: Lung Image Database Consortium, BreakHis: Breast Cancer Histopathology Database, ROI: Region of Interest, MCC: Matthew’s Correlation Coefficient, mp-MRI: MultiParametric Magnetic Resonance Imaging, AUC: Area Under the Curve) | | | | | | | | | | | | |

| **Table 2: Published works that implemented the decision tree and random forest models in cancer research.** | | | | | | | | | | | |
| --- | --- | --- | --- | --- | --- | --- | --- | --- | --- | --- | --- |
| Ref | Year | Dataset | Cancer Type | Number of Patients | Method Name | Input | Data Preprocessing Technique | Clinical Endpoint | Model Validation Technique | Results |  |
| [81] | 2003 | NA | Ovarian | 139 | Decision tree | Serum samples * | SELDI processing of the serum samples | Malignancy level (malignant or benign) | 10-fold cross-validation | Sensitivity: 80%  Specificity: 80% |  |
| [82] | 2006 | NA | Gastric | 245 | Decision tree | Serum samples * | SELDI processing of the serum samples | Malignancy level (malignant or benign) | 10-fold cross-validation | Accuracy: 86.4%  Sensitivity: 85.3%  Specificity: 88% |  |
| [83] | 2009 | SEER breast cancer incidence data in the years 1973–2004. ([Link](https://seer.cancer.gov/)) | Breast | 779,999 | Decision tree | age, sex, race, grade, SEER stage, tumor size, lymph node involvement, degree of extension, surgical  intervention | 1)Data Cleaning  2) Dimensionality reduction  3) Feature engineering | Survivability | 10-fold stratified cross-validation | AUC: 60.7%  Accuracy: 88.05%  Sensitivity: 98.14%  Specificity: 23.25% |  |
| [84] | 2019 | WDBC ([Link](https://archive.ics.uci.edu/ml/datasets/breast+cancer+wisconsin+(diagnostic))) | Breast | 569 | Decision tree | Id, diagnosis, radius, texture, perimeter, area, concavity, smoothness, compactness, mean concave points, symmetry, mean fractal dimension | NA | Malignancy level (malignant or benign) | NA | Accuracy: 99% |  |
| [85] | 2019 | UCI-WBC dataset ([Link](https://archive-beta.ics.uci.edu/dataset/15/breast+cancer+wisconsin+original)) | Breast | 699 | Random forest | Clump Thickness, Uniformity of Cell Size, Uniformity of Cell Shape, Marginal Adhesion, Single Epithelial Cell Size, Bare Nuclei, Bland Chromatin, Normal Nucleoli, Mitoses | NA | Malignancy level (malignant or benign) | NA | Accuracy > 99% |  |
| [86] | 2019 | NA | Prostate | NA | Random forest | NA | NA | Malignancy level / susceptibility | NA | NA |  |
| [87] | 2012 | Microarray Dataset ([Link](http://genomics-pubs.princeton.edu/oncology/affydata/index.html)) | Colon | 62 | Random forest | NA | NA | Susceptibility | NA | Sensitivity: 83.75%  Specificity: 76.15% |  |
| [88] | 2019 | SEER breast cancer incidence data in the years 1973–2015 ([Link](https://seer.cancer.gov/)) | Breast | 122,809 | Random forest | age, sex, race, grade, SEER stage, tumor size, lymph node involvement, degree of extension, surgical  intervention | NA | Recurrence | NA | NA |  |
| [89] | 2014 | NA | prostate | 261 | Random forest | histograms | NA | Treatment responses | NA | NA |  |

(SELDI: Surface-Enhanced Laser Desorption/Ionization, SEER: Surveillance, Epidemiology, and End Results, WDBC: Wisconsin Diagnostic Breast Cancer, WBC: Wisconsin Breast Cancer)

* Original sources referred to in these studies provided only general information about serum samples without specifying the markers or components involv

| **Table 3: Published works that implemented the K-Nearest Neighbors (KNN) model in cancer research.** | | | | | | | | | | | |
| --- | --- | --- | --- | --- | --- | --- | --- | --- | --- | --- | --- |
| Ref | Year | Dataset | Cancer Type | Number of Patients | Method Name | Input | Data Preprocessing Technique | Clinical Endpoint | Model Validation Technique | Results |  |
| [90] | 2016 | NA | Cervical | NA | KNN | Pap smear images | 1) De-noising  2) edge-based segmentation  3) feature extraction (such as Area, Elongation, and Perimeter of cell nuclei  and cytoplasm and finally the N/C ratio) | Malignancy Level (malignant or benign) | 5-fold cross-validation | Accuracy: 82.9% |  |
| [91] | 2013 | WDBC ([Link](https://archive.ics.uci.edu/ml/datasets/breast+cancer+wisconsin+(diagnostic))) | Breast | 699 | KNN | NA | NA | Malignancy Level (malignant or benign) | NA | Accuracy: 98.7% |  |
| [92] | 2012 | NA | Gastric | 38 | KNN | GSI | 1) ROI segmentation  2) Feature extraction  3) Normalization  4) Feature Selection | Malignancy Level (malignant or benign) | NA | Accuracy: 96.33% |  |
| [93] | 2018 | Mini-MIAS database | Breast | 61 | KNN | Mammographic images | 1) De-noising  2) segmentation, Otsus thresholding  3) GLCM feature extraction (mean, standard deviation, skewness, energy, contrast, correlation, homogeneity, entropy) | Malignancy Level (malignant or benign) | NA | Accuracy: 92% |  |
| [94] | 2021 | Breast Cancer ([Link](https://web.inf.ufpr.br/vri/databases/breast-cancer-histopathological-database-breakhis/))  breast histopathology images from Kaggle ([Link](https://www.ncbi.nlm.nih.gov/pubmed/)) | Breast | - 82  - 162 | KNN-BCD | Histopathologic images | 1) texture feature extraction  2) dimensionality reduction using LDA | Malignancy Level (malignant or benign) | NA | Accuracy: 80%  Sensitivity: 81.81%  Specificity: 78.57%  Selectivity: 75%  F-score: 12% |  |

(KNN: K-Nearest Neighbors, WDBC: Wisconsin Diagnostic Breast Cancer, GSI: Gemstone Spectral Imaging, MRI: Magnetic Resonance Imaging, MIAS: Mammographic Image Analysis Society, GLCM: Gray Level Co-occurrence Matrix)

| **Table 4: Published works that have implemented the K-means model in cancer research.** | | | | | | | | | |  |
| --- | --- | --- | --- | --- | --- | --- | --- | --- | --- | --- |
| Ref | Year | Dataset | Cancer Type | Number of Patients | Method Name | | Input | Clinical Endpoint | Results | |
| [95] | 2016 | UCI-WBC dataset ([Link](https://archive-beta.ics.uci.edu/dataset/15/breast+cancer+wisconsin+original)) | Breast | 675 | | K-means | Clump Thickness, Uniformity of Cell Size, Uniformity of Cell Shape, Marginal Adhesion, Single Epithelial Cell Size, Bare Nuclei, Bland Chromatin, Normal Nucleoli, Mitoses | Malignancy Level (malignant or benign) | Average Positive Prediction Accuracy): 92% | |
| [96] | 2013 | NA | Lung | 177 | | Foggy K-means | Age, sex, BMI, family history, tuberculosis, smoking, lymph node involvement, tumor size, radiation/radon/asbestos | Malignancy Level (malignant or benign) | Dunn Index: 0.1616  Silhouette: 0.5136  Connectivity: 9.842 | |
| [97] | 2010 | WRBC ([Link](http://archive.ics.uci.edu/ml/machinelearning-databases/breast-cancer-wisconsin/)) | Breast | 198 | | K-means | Radius, center, texture, perimeter, area, smoothness, compactness, concavity, concave points, fractal dimension, tumor size, lymph node status | Recurrence (non-recurrence-events, recurrence-events) | NA | |

(WBC: Wisconsin Breast Cancer, BMI: Body Mass Index, WRBC: Wisconsin Recurrence Breast Cancer database)

| **Table 5: Published works that have implemented the logistic regression model in cancer research.** | | | | | | | |
| --- | --- | --- | --- | --- | --- | --- | --- |
| Ref | Year | Dataset | Cancer Type | Number of Patients | Input | Clinical Endpoint | Results |
| [98] | 2018 | WDBC ([Link](https://archive.ics.uci.edu/ml/datasets/breast+cancer+wisconsin+(diagnostic))) | Breast | 569 | maximum texture and maximum perimeter | Malignancy Level (malignant or benign) | Accuracy: 96.5 |
| [99] | 2018 | NA | Lung | 475 | Age, Gender, Height, Weight, and Smoking history  … | Malignancy Level (malignant or benign) | Sensitivity: 96.2%  Specificity: 90.6% |
| [100] | 2015 | WDBC ([Link](https://archive.ics.uci.edu/ml/datasets/breast+cancer+wisconsin+(diagnostic))) | Breast | 569 | Texture, Perimeter, Area, Concavity, Symmetry | Malignancy Level (malignant or benign) | Accuracy: 98.9%  Sensitivity: 98.5%  Specificity: 99.1% |
| (WDBC: Wisconsin Diagnostic Breast Cancer) | | | | | | | |

| **Table 6: Published works that have implemented the**  **Naive Bayes model in cancer research.** | | | | | | | | | | | | |  |
| --- | --- | --- | --- | --- | --- | --- | --- | --- | --- | --- | --- | --- | --- |
| Ref | Year | Dataset | Cancer Type | Number of Patients | | Input | Data Preprocessing Technique | | | Clinical Endpoint | | Results |  |
| [101] | 2019 | NA | Colon | 209 | Age, CEA, hemoglobin  (gram/dL), Leukocytes  (cell/mm3), Haematocrit  (mL/L), Thrombocyte  (cell/mm3) | | | NA | Malignancy Level (malignant or benign) | | Accuracy: 95.24%  Precision: 100%  F1-score: 96% | | |
| [102] | 2019 | Rembrandt database ([Link](https://wiki.cancerimagingarchive.net/display/Public/REMBRANDT)) | Brain | 114 | MRI images | | | 1) grey-scale conversion  2) morphological opening  3) Pixel subtraction  4) segmentation (maximum entropy)  5) feature extraction (region features: area,  perimeter, eccentricity, equivalent diameter, solidity, and convex  area, major axis length, and minor axis length.  Features: maximum, mean, and minimum) | Malignancy Level (malignant or benign) | | Accuracy: 94%  Sensitivity: 81.25%  Specificity: 100% | | |
| [103] | 2015 | UCI-WBC dataset ([Link](https://archive-beta.ics.uci.edu/dataset/15/breast+cancer+wisconsin+original)) | Breast | 683 | Clump Thickness, Uniformity of Cell Size, Uniformity of Cell Shape, Marginal Adhesion, Single Epithelial Cell Size, Bare Nuclei, Bland Chromatin, Normal Nucleoli, Mitoses | | | NA | Malignancy Level (malignant or benign) | | Success rate: 94% | | |
| [104] | 2012 | UCI-WBC dataset ([Link](https://archive-beta.ics.uci.edu/dataset/15/breast+cancer+wisconsin+original)) | Breast | 699 | Clump Thickness, Uniformity of Cell Size, Uniformity of Cell Shape, Marginal Adhesion, Single Epithelial Cell Size, Bare Nuclei, Bland Chromatin, Normal Nucleoli, Mitoses | | | NA | Malignancy Level (malignant or benign) | | Accuracy: 97,5%  Precision: 100%  Sensitivity: 96,7%  Selectivity:73.8%  Specificity: 100% | | |
| [105] | 2017 | NA | Lung | 35 | Microscopic lung biopsy images | | | 1) grey-scale conversion  2) texture feature extraction (contrast, correlation, energy, homogeneity) | Malignancy Level (malignant or benign) | | Accuracy: 88.57% | | |

| **Table 7: Published works that implemented the eXtreme Gradient Boosting (XGBoost) model in cancer research.** | | | | | | | | |  |
| --- | --- | --- | --- | --- | --- | --- | --- | --- | --- |
| Ref | Year | Dataset | Cancer Type | Number of Patients | Data Preprocessing Technique | Clinical Endpoint | Model Validation Technique | Results | |
| [106] | 2019 | CRlncRC ([Link](https://github.com/xuanblo/CRlncRC)) | NA | NA | 1) Data preparation (feature extraction, data cleaning)  2) feature engineering (Laplacian score calculation, feature integration, normalization) | Susceptibility | 10-fold cross-validation | Precision: 90%  Recall: 71%  F1-score: 74% | |
| [107] | 2020 | NA | Lung | 81 | NA | Malignancy Level (malignant (stage I, stage II, stage III, stage IV) or benign) | 5-cross validation | Sensitivity: 100%  Specificity: 100% | |
| (CRlncRC: Cancer-Related Long Non-Coding RNAs, XGBoost: eXtreme Gradient Boost) | | | | | | | | |  |

| **Table 8: The published works that have implemented hybrid models in cancer research.** | | | | | | | | | | | |
| --- | --- | --- | --- | --- | --- | --- | --- | --- | --- | --- | --- |
| Ref | Year | Dataset | Cancer Type | Number of Patients | Method Name | Input | Data Preprocessing Technique | Clinical Endpoint | Model Validation Technique | Results |  |
| [10] | 2018 | NA | Breast | 822 | SOM, CVNN | Shape of mass, density of mass, margin of mass, patient’s age, BI-RADS assessment | Min-Max normalization | Malignancy Level (malignant or benign) | NA | Sensitivity: 0,95  Specificity: 0.94  Accuracy: 0,945  ROC: 0,93 |  |
| [108] | 2022 | NA | Breast | 116 | SVM, extra-trees | BMI, age, glucose levels, homeostasis model  assessment, insulin, adiponectin, leptin, MCP-1, resistin | 1) Data Normalization  2) Feature selection using the extra-trees method | Malignancy Level (malignant or benign) | 10-fold cross-validation | Sensitivity: 78.57%  Specificity: 78.57%  Accuracy: 80.23%  Precision: 82.71%  AUC: 0 ,78 |  |
| [109] | 2019 | NA | Lung | NA | SVM, KNN | CT scan image | 1) Noise removal  2) contrast expansion using CLAHE equalization  3) ROI Segmentation  4) Feature extraction using the GLCM | Malignancy Level (malignant or benign) | NA | NA |  |
| [110] | 2019 | UCI-WBC dataset ([Link](https://archive-beta.ics.uci.edu/dataset/15/breast+cancer+wisconsin+original)) | Breast | 699 | PCA, ANN | Radius [mean of distances from the center to points on the perimeter], Texture [standard deviation of grey-scale values], Perimeter, Area, Smoothness [local variation in radius lengths], Compactness, Concavity [severity of concave portions of the contour], Concave points, Symmetry, Fractal dimension ["coastline approximation" - 1] | 1) handling missing data  2) dimensionality reduction (feature selection) using PCA | Malignancy Level (malignant or benign) | 10-fold cross-validation | Accuracy: 0.97  Sensitivity: 0.95  Specificity: 0.98 |  |
| [111] | 2019 | LIDC ([Link](https://wiki.cancerimagingarchive.net/pages/viewpage.action?pageId=1966254)) | Lung | 455 | SVM, KNN | CT scan images | 1) Image pre-processing  2) Segmentation of the lung region  3) Feature extraction (geometric, texture, and statistical features)  4) Feature selection | Malignancy Level (malignant or benign) | NA | Accuracy: 97.6%  Sensitivity: 98,9  Specificity: 95.2  Precision: 97.4 |  |
| [112] | 2021 | WDBC ([Link](https://archive.ics.uci.edu/ml/datasets/breast+cancer+wisconsin+(diagnostic))) | Breast | 569 | Genetic search + KNN, multilayer perceptron | Radius, texture, perimeter, area, smoothness, compactness, concavity, concave points, symmetry, fractal dimension | 1) Data pre-processing  2) Data imbalance handling  3) Feature selection | Malignancy Level (malignant or benign) | NA | Accuracy: 98.6%  Sensitivity: 98.6%  Specificity: 98.6%  AUC: 0,99  MCC: 0.97 |  |
| [113] | 2013 | WDBC ([Link](https://archive.ics.uci.edu/ml/datasets/breast+cancer+wisconsin+(diagnostic))) | Breast | 569 | K-means, SVM | Radius, texture, perimeter, area, smoothness, compactness, concavity, concave points, symmetry, fractal dimension | Feature selection | Malignancy Level (malignant or benign) | 10-fold cross-validation | Accuracy: 97.38 |  |
| (SOM: Self-Organizing Map, CVNN: Complex-Valued Neural Network, AUC: Area Under the Curve, SVM: Support Vector Machine, BMI: Body Mass Index, KNN: K-Nearest Neighbors, CT Scan: Computed Tomography Scan, WBC: Wisconsin Breast Cancer, PCA: Principal Component Analysis, ANN: Artificial Neural Network, LIDC: Lung Image Database Consortium, WDBC: Wisconsin Diagnostic Breast Cancer) | | | | | | | | | | |  |

| **Table 9: Published works that have implemented the fuzzy inference system (FIS) model in cancer research.** | | | | | | | | | | | |
| --- | --- | --- | --- | --- | --- | --- | --- | --- | --- | --- | --- |
| Ref | Year | Dataset | Cancer Type | Number of Patients | Method name | Important Features | The type of Machine Learning Problem | Data Preprocessing Technique | Clinical Endpoint | Model Validation Technique | Results |
| [114] | 2021 | TCGA ([Link](https://www.cancer.gov/ccg/research/genome-sequencing/tcga)) | kidney | 1249 | self-organizing deep neuro-fuzzy system | A subset of 60 miRNAs | Classification | Data Transformation and Dimensionality Reduction | Classifying kidney cancer subtypes | Hold-out | 93.2% Accuracy |
| [115] | 2016 | UCSB | Breast | 50 | FIS | Area, compactness, eccentricity,  major axis length, mean radius, and perimeter | Classification | Cropping, Unmix, Unmix to color and color to gray, thresholdingand segmentation | Benign/malignant | Not detailed | 94.26% Accuracy |
| [116] | 2020 | Private | Renal | 200 | ANFIS | smoking, dialysis, occupational  exposure (OE), genetic or hereditary (GOR)and hematuria, RBCC, FP, TS, VHLG, HBP, TCEE | Classification | Not detailed | cancer/no cancer & cancer stages | 4-fold cross-validation | 98% Accuracy |
| [117] | 2015 | Private | esophageal | 271 | ANFIS | CRP, albumin, time interval, AJCC stage, and treatment method | Regression | Data Transformation and Expansion | Survivability | Hold-out | Not detailed |
| [118] | 2020 | Hospital | lung | 100 | FSES | Weight loss,  Shortness of breath, chest pain, blood in sputum, persistent cough, age | Classification | Dimensionality Reduction | cancer/No cancer | Hold-out | 100% quantized accuracy |
| [119] | 2015 | DDSM ([Link](http://www.eng.usf.edu/cvprg/mammography/database.html)) | Breast | 36 | FIS | Shape, contour, density | Classification | Not detailed | malignancy degree | Hold-out | 83.34% Accuracy |
| [120] | 2018 | mini-MIAS ([Link](http://peipa.essex.ac.uk/info/mias.html)) | Breast | 322 | FIS | Compactness, entropy, mean,  and smoothness | Classification | Removal of labels and noise, heightening of contrast, and ROI segmentation | Normal/abnormal | 10-fold cross-validation | 89.37±6.62% Accuracy |
| [121] | 2020 | 1. BRATS 12  2.BRATS13  ([Link](http://www2.imm.dtu.dk/projects/BRATS2012/data.html)) | Brain | 45(BRATS 12),  65(BRATS 13) | T2FIS-ANFIS | Similarity, dilation, midline shift, mass effect, circularity, contrast | Classification | De-noising, removal of skull, segmentation, normalization | Benign/malignant | 5-fold cross-validation | BRATS 12: 91.2% accuracy, BRATS 13: 90.9% accuracy |
| [122] | 2022 | Private laboratory and clinical | prostate | 100 | FIS | Age, prostate volume (PV), prostate-specific antigen (PSA), free prostate- specific  Antigen (FPSA) | Regression | Not detailed | Risk assessment | Not detailed | Not detailed |
| [123] | 2017 | 1. WBCD ([Link](http://archive.ics.uci.edu/ml/datasets/breast+cancer+wisconsin+(original)))  2. Mammographic mass | Breast | WBCD:569,  Mammographic mass: 961 | EM-PCA-CART-Fuzzy Rule-Based | WBCD: Radius, texture, perimeter, area, smoothness, compactness, concavity,  Concave points, Symmetry, Fractal dimension  Mammographic mass: BI-RADS, age, shape, margin, density | Classification | Not detailed | Benign/malignant | 10-fold cross-validation | WBCD: 93.2% accuracy, mammographic mass: 94.1% accuracy |

(TCGA: The Cancer Genome Atlas, miRNA: Micro-ribonucleic Acids, UCSB: University of California, Santa Barbara, ANFIS: Adaptive Network-based Fuzzy Inference System, RBCC: Red Blood Cell Count, FP: Flank Pain, TS: Tumour Size, VHLG: Von Hippel-Lindau Gene, HBP: High Blood Pressure, TCEE: Trichloroethylene Exposure, CRP: C-Reactive Protein, AJCC: American Joint Committee on Cancer, FSES: Fuzzy Soft Expert System, DDSM: Digital Database for Screening Mammography, MIAS: Mammographic Image Analysis Society, BRATS: Multimodal Brain Tumour Image Segmentation Benchmark, WBCD: Wisconsin Breast Cancer Database, T2FIS: Type-II Fuzzy Inference System, EM: Expectation Maximization, PCA: Principal Component Analysis, CART: Classification and Regression Trees, BI-RADS: Breast Imaging Reporting and Data System)

| **Table 10: Published works that have implemented hybrid soft computing algorithms in cancer research.** | | | | | | | | | | | |
| --- | --- | --- | --- | --- | --- | --- | --- | --- | --- | --- | --- |
| Ref | Year | Dataset | Cancer Type | Number of Patients | Method name | Important Features | The type of Machine Learning Problem | Data Preprocessing Technique | Clinical Endpoint | Model Validation Technique | Results |
| [124] | 2022 | ISIC2019 ([Link](https://www.kaggle.com/datasets/andrewmvd/isic-2019)) | Skin | 25,331 | ODNN-IWOA | Dermoscopic findings | Classification | Removal of noise and segmentation | Classification of skin cancer subtypes | Holdout | 99.90% Accuracy |
| [125] | 2015 | WBCD ([Link](http://archive.ics.uci.edu/ml/datasets/breast+cancer+wisconsin+(original))) | Breast | 699 | GONN | FNA findings | classification | Data cleaning | Benign/malignant | 10-fold cross-validation | 100% Accuracy |
| [126] | 2023 | BreakHis ([Link](https://www.kaggle.com/datasets/ambarish/breakhis)) | Breast | 1174 | CNN-PSO-ASO-EO | Histopathological findings | classification | Dimensionality Reduction | Benign/malignant | Holdout | 97.73% Accuracy |
| [127] | 2023 | Private | esophageal | 299 | GA-DNN | TNM, WBC, FIB, PT,  Age, final degree of differentiation, APTT, TT, final length, ALB, INR, PLT, RBC, and GLB | Regression | Dimensionality Reduction | Survivability | 5-fold cross-validation | 91% AUC |
| [128] | 2019 | Hospital | cervical | 799 | HMLP-GA | size of nucleus, size of cytoplasm, gray level of nucleus, and gray level of cytoplasm | Classification | Not detailed | categorizing Normal/LSIL/HSIL | 5-fold cross-validation | 74.82% Accuracy |
| [129] | 2021 | Hospital | Liver | 10060 | PSO-ELM | TCM symptoms, signs, tongue diagnostic information, and pulse  Diagnostic information | Classification | Dimensionality Reduction | Classifying liver cancer  Syndromes | Holdout | 86.26%  Accuracy |
| [130] | 2016 | WDBC ([Link](https://archive.ics.uci.edu/ml/datasets/breast+cancer+wisconsin+(diagnostic))),  WBCD ([Link](http://archive.ics.uci.edu/ml/datasets/breast+cancer+wisconsin+(original))) | Breast | WDBC:569  WBCD:699 | PSO-KDE | FNA findings | Classification | Dimensionality Reduction | Benign/malignant | 10-fold cross-validation | WDBC: 98.45% accuracy  WBCD: 98.53 % Accuracy |
| [131] | 2021 | DDSM ([Link](http://www.eng.usf.edu/cvprg/mammography/database.html)),  WBC ([Link](https://archive.ics.uci.edu/ml/datasets/breast+cancer+wisconsin+(diagnostic))) | Breast | DDSM:651  WBC:569 | DDSM: BOAALO-ANFIS, WBC: BOAALO-ANN | intensity, texture (extracted by GLCM), and shape-based features | Classification | Removal of noise, labels, and segmentation | Benign/malignant | Holdout | DDSM: 98.50% accuracy  WBC: 98.16 % accuracy |
| [132] | 2022 | Herlev ([Link](http://mde-lab.aegean.gr/index.php/downloads)) | Cervical | 917 | GA-MLP | texture-based features extracted using MULTP | Classification | segmentation | Normal/abnormal | 5-fold and 10-fold cross-validation | 5-fold: 98.90%  Accuracy,  10-fold: 98.80%  Accuracy, |

(ISIC: International Skin Imaging Collaboration, ODNN: Optimal Deep Neural Network, IWOA: Improved Whale Optimization Algorithm, WBCD: Wisconsin Breast Cancer Database, GONN: Genetically Optimized Neural Network, FNA: Fine Needle Aspiration, BreakHis: Breast Cancer Histopathology Database, CNN: Convolutional Neural Network, PSO: Particle Swarm Optimization, ASO: Atom Search Optimization, EO: Equilibrium Optimizer, GA: Genetic Algorithm, DNN: Deep Neural Network, TNM: Tumour (T), Node (N), Metastasis (M), WBC: White Blood Cells, FIB: Fibrinogen, PT: Prothrombin Time, APTT: Activated Partial Thromboplastin Time, TT: Thrombin Time, ALB: Albumin, INR: International Normalized Ratio, PLT: Platelet Count, RBC: Red Blood Cells, GLB: Globulin, AUC: Area Under the Curve, HMLP: Hybrid Multi-layered Perceptron, GA: Genetic Algorithm, TCM: Traditional Chinese Medicine, PSO: Particle Swarm Optimization, ELM: Extreme Learning Machine, WDBC: Wisconsin Diagnostic Breast Cancer, DDSM: Digital Database for Screening Mammography, BOAALO: Butterfly Optimization Algorithm and Ant Lion Optimizer, ANFIS: Adaptive Network-based Fuzzy Inference System, ANN: Artificial Neural Network, MLP: Multi-layer Perceptron)

| **Table 11: Published works that have implemented deep learning in cancer research.** | | | | | | | | | | | | | | | | | | | | |  |
| --- | --- | --- | --- | --- | --- | --- | --- | --- | --- | --- | --- | --- | --- | --- | --- | --- | --- | --- | --- | --- | --- |
| Ref | Year | Dataset | Cancer Type | | Number of Patients | | Method | | Important Features | | The type of Machine Learning Problem | | Data Preprocessing Technique | | Clinical Endpoint | | Model Validation Technique | | Results | |  |
| [133] | 2023 | MIAS ([Link](https://www.repository.cam.ac.uk/handle/1810/250394)) | Breast | | 322 | | CNN+BiLSTM | | mammography findings | | Classification | | Removal of noise and defects, segmentation, and cropping | | Normal/Benign/ malignant | | Hold-out | | 98.56% Accuracy | |  |
| [134] | 2023 | BreakHis ([Link](https://www.kaggle.com/datasets/ambarish/breakhis)) | Breast | | 7909 | | CNN | | Histopathological findings | | Classification | | Resizing, noise reduction, and improving the  brightness | | Benign/malignant | | Hold-out | | 99.52±0.24% Accuracy | |  |
| [135] | 2022 | Lung-PET-CT-Dx ([Link](https://www.cancerimagingarchive.net/)) | Lung | | 355 | | Dense CNN+CSE | | CT image findings | | Classification | | Resizing, normalization, data augmentation, and labeling | | Classifyinglung cancer stages | | 5-fold cross-validation | | 97% Accuracy | |  |
| [136] | 2023 | Private medical center | prostate | | 2187 | | Multimodal CNN | | SERS, age, PSA levels,and pathological results | | Classification | | Not detailed | | PCa/BPH | | simple cross-validation | | 88.55 ± 0.66% accuracy | |  |
| [137] | 2021 | HUAP | Breast | | 287 | | Multi-input CNN | | Personal and clinical features and thermal imaging findings | | Classification | | Data cleansing and normalization | | Healthy/sick | | 5-fold cross-validation | | 97% Accuracy | |  |
| [138] | 2023 | Hospital | Breast | | 550 | | Deep CNN | | cytological findings | | Classification | | Segmentation, normalization, and patching | | Benign/malignant | | Hold-out | | 94.55% Accuracy | |  |
| [139] | 2022 | BreakHis ([Link](https://www.kaggle.com/datasets/ambarish/breakhis)) | Breast | | 7909 | | Deep CNN+TL | | Histopathological findings | | Classification | | Resizing, noise reduction, and data augmentation | | Benign/malignant | | Hold-out | | ResNet: 99.7% accuracy,  Inception-V3Net:  97.66% Accuracy  ShuffleNet: 96.94% accuracy | |  |
| [140] | 2022 | Local clinics and oncology dispensaries | Skin | | 617 | | CNN | | Raman spectral findings | | classification | | Excluding background signals and noise | | Benign/malignant | | 10-fold cross-validation | | 96% AUC | |  |
| [141] | 2020 | Herlev ([Link](http://mde-lab.aegean.gr/index.php/downloads)) | Cervical | | 917 | | CNN-ELM | | Cell image findings | | classification | | Not detailed | | normal/abnormal | | 5-fold cross-validation | | 99.5% accuracy (2-class), 91.2% accuracy (7-class) | |  |
| [142] | 2022 | BreakHis ([Link](https://www.kaggle.com/datasets/ambarish/breakhis)), ICIAR ([Link](https://iciar2018-challenge.grand-challenge.org/Dataset/)) | Breast | | BreakHis: 7909, ICIAR: 400 | | multi-scale DNN | | Histopathological findings | | classification | | Resizing, data augmentation, and vertical and horizontal flipping | | Benign/ malignant and normal/benign/in situ invasive | | Hold-out | | BreakHis: 99.2% accuracy, ICIAR: 95.2% accuracy | |  |
| [143] | 2017 | Private | Breast | | 1874 | | Deep CNN | | mammography findings | | Classification | | Wavelet transform | | Benign/malignant | | Random sub-sampling | | 82.43% Accuracy | |  |
| [144] | 2019 | ICIAR ([Link](https://iciar2018-challenge.grand-challenge.org/Dataset/)) | Breast | | 400 | | EMS-Net | | Histopathological findings | | Classification | | Resizing and patching | | Normal/benign/in situ invasive | | 5-fold cross-validation | | 91.75 ±2.32% accuracy | |  |
| [145] | 2023 | BUSI ([Link](https://www.kaggle.com/datasets/aryashah2k/breast-ultrasound-images-dataset)) | Breast | | 780 | | Fuzzy ensemble-based model | | Ultrasound imagingfindings | | Classification | | Resizing | | Benign/malignant/normal | | 5-fold cross-validation | | 85.23 ± 2.52% accuracy | |  |
| [146] | 2023 | Hospital | Breast | | 290 | | TAI-net | | ADC and DWI images findings | | Classification | | Resizing, padding, and data augmentation | | Benign/malignant | | 5-fold cross-validation | | 89.0 ±1.5% accuracy | |  |
| [147] | 2023 | BUSI1311 ([Link](https://www.kaggle.com/datasets/aryashah2k/breast-ultrasound-images-dataset)) | Breast | | 500 | | BEiT-RNN-LSTM | | Ultrasound imagefindings | | Classification | | Normalization, patching, noise reduction, and image enhancement | | Normal/Benign/ malignant | | Hold-out | | 99% Accuracy | |  |
| [148] | 2023 | ETIS-Larib ([Link](http://www.cvc.uab.es/CVC-Colon/index.php/databases/)),  CVC-VideoClinicDB ([Link](http://www.cvc.uab.es/CVC-Colon/index.php/databases/)),  CVC-EndoSceneStill ([Link](http://www.cvc.uab.es/CVC-Colon/index.php/databases/)),  Kvasir-SEG ([Link](https://datasets.simula.no/kvasir-seg/)), SUN Colonoscopy Video Database ([Link](http://sundatabase.org/)),  CVC-Segementation-HD ([Link](http://www.cvc.uab.es/CVC-Colon/index.php/databases/)),  Endoscopy Disease Detection Challenge 2020 ([Link](https://endocv2022.grand-challenge.org/Data/)) | | Colorectal | | 506,336 | | Deep CNN | | Colonoscopy findings | | Classification | | Resizing, Normalization, and Data augmentation | | Classifying colorectal cancer stages | | 5-fold cross-validation | | Precision 99.71, Recall 87.05, F1 92.95 | |
| [149] | 2018 | Hospital | | Pancreatic | | 219 | | ANN | | Age, sex, resection, adjunctive therapy, stage, duration, and eight SF-36 domain values | | Regression | | Not detailed | | Survivability | | 2-fold cross-validation | | Accuracy>70%,  Sensitivity>91% | |
| [150] | 2021 | SEER ([Link](https://seer.cancer.gov/)) | | Bladder | | 161,227 | | ANN | | age, sex, race, grade, SEER stage, tumor size, lymph node involvement, degree of extension, surgical  intervention | | Regression | | Not detailed | | Survivability | | Hold-out | | OS model: 81% AUC  DSS model: 81% AUC | |
| [151] | 2022 | Hospital | | Breast | | 1844 | | ANN | | Age, laterality, Education Status, Comorbidities,  CVC history, chemotherapy status, insertion attempts, and upper extremity activity | | Classification | | Not detailed | | Occurrence of thrombosis | | Hold-out | | 72.5% AUC | |
| [152] | 2023 | Hospital | | Breast | | 74 | | PLS-ANNDA | | immunohistochemical findings from the tumor tissue | | classification | | Normalization | | Classifying themolecular subtypes of breast cancer | | 15-fold Venetian blind cross-validation | | 100% accuracy | |
|  |  |  | |  | |  | |  | |  | |  | |  | |  | |  | |  | |

(MIAS: Mammographic Image Analysis Society, CNN: Convolutional Neural Network, BILSTM: Bidirectional Long Short-Term Memories, BreakHis: Breast Cancer Histopathology Database, Lung-PET-CT-Dx: A Large-Scale CT and PET/CT Dataset for Lung Cancer Diagnosis, CSE: Concurrent Squeeze & Excitation, CT: Computed Tomography, SERS: Surface-enhanced Raman Spectroscopy, PSA: Prostate Specific Antigen, PCa: Prostate Cancer, BPH: Benign Prostatic Hyperplasia, HUAP: University Hospital Antônio Pedro, TL: Transfer Learning, AUC: Area Under the Curve, ELM: Extreme Learning Machine, EMS-Net: Ensemble of MultiScale convolutional neural Network, TAI-Net: Triple-attention interaction network, ADC: Apparent Dispersion Coefficient, DWI: Diffusion-weighted Imaging, BEiT: BERT Pre-training of Image Transformers, RNN: Recurrent Neural Network, LSTM: Long Short-Term Memory, ANN: Artificial Neural Network, SEER: Surveillance, Epidemiology and End Results, AUC: Area Under the Curve, DSS: Disease Specific Survival, , CVC: Central Venous Catheter, PLS-ANNDA: Partial Least Square-artificial Neural Network Discriminant Analys

**References**

69. Rejani, Y. and S.T. Selvi, *Early detection of breast cancer using SVM classifier technique.* arXiv preprint arXiv:0912.2314, 2009.

70. Vijayarajeswari, R., P. Parthasarathy, S. Vivekanandan, and A.A. Basha, *Classification of mammogram for early detection of breast cancer using SVM classifier and Hough transform.* Measurement, 2019. **146**: p. 800-805.

71. Maglogiannis, I., E. Zafiropoulos, and I. Anagnostopoulos, *An intelligent system for automated breast cancer diagnosis and prognosis using SVM based classifiers.* Applied intelligence, 2009. **30**: p. 24-36.

72. Asuntha, A., A. Brindha, S. Indirani, and A. Srinivasan, *Lung cancer detection using SVM algorithm and optimization techniques.* J. Chem. Pharm. Sci, 2016. **9**(4): p. 3198-3203.

73. Xu, G., M. Zhang, H. Zhu, and J. Xu, *A 15-gene signature for prediction of colon cancer recurrence and prognosis based on SVM.* Gene, 2017. **604**: p. 33-40.

74. Osman, A.H., *An enhanced breast cancer diagnosis scheme based on two-step-SVM technique.* International Journal of Advanced Computer Science and Applications, 2017. **8**(4).

75. Jabber, B., et al. *SVM model based computerized bone cancer detection*. in *2020 4th International Conference on Electronics, Communication and Aerospace Technology (ICECA)*. 2020. IEEE.

76. Kaucha, D.P., et al. *Early detection of lung cancer using SVM classifier in biomedical image processing*. in *2017 IEEE International Conference on Power, Control, Signals and Instrumentation Engineering (ICPCSI)*. 2017. IEEE.

77. Singh, S. and R. Kumar. *Histopathological image analysis for breast cancer detection using cubic SVM*. in *2020 7th international conference on signal processing and integrated networks (SPIN)*. 2020. IEEE.

78. Qayyum, A. and A. Basit. *Automatic breast segmentation and cancer detection via SVM in mammograms*. in *2016 International conference on emerging technologies (ICET)*. 2016. IEEE.

79. Bhattacharjee, S., et al., *Quantitative analysis of benign and malignant tumors in histopathology: Predicting prostate cancer grading using SVM.* Applied Sciences, 2019. **9**(15): p. 2969.

80. Li, J., et al., *Support Vector Machines (SVM) classification of prostate cancer Gleason score in central gland using multiparametric magnetic resonance images: A cross-validated study.* European journal of radiology, 2018. **98**: p. 61-67.

81. Xu, Q. and K.S. Lam, *Protein and chemical microarrays—powerful tools for proteomics.* Journal of Biomedicine and Biotechnology, 2003. **2003**(5): p. 257.

82. Su, Y., et al., *Diagnosis of gastric cancer using decision tree classification of mass spectral data.* Cancer science, 2007. **98**(1): p. 37-43.

83. Liu, Y.-Q., C. Wang, and L. Zhang. *Decision tree based predictive models for breast cancer survivability on imbalanced data*. in *2009 3rd international conference on bioinformatics and biomedical engineering*. 2009. IEEE.

84. Sathiyanarayanan, P., S. Pavithra, M.S. Saranya, and M. Makeswari. *Identification of breast cancer using the decision tree algorithm*. in *2019 IEEE International conference on system, computation, automation and networking (ICSCAN)*. 2019. IEEE.

85. Octaviani, T. and d.Z. Rustam. *Random forest for breast cancer prediction*. in *AIP Conference Proceedings*. 2019. AIP Publishing LLC.

86. Toth, R., et al., *Random forest-based modelling to detect biomarkers for prostate cancer progression.* Clinical epigenetics, 2019. **11**: p. 1-15.

87. Yan, Z., et al., *Identification of candidate colon cancer biomarkers by applying a random forest approach on microarray data.* Oncology reports, 2012. **28**(3): p. 1036-1042.

88. Imani, F., R. Chen, C. Tucker, and H. Yang. *Random forest modeling for survival analysis of cancer recurrences*. in *2019 IEEE 15th International Conference on Automation Science and Engineering (CASE)*. 2019. IEEE.

89. Ospina, J.D., et al., *Random forests to predict rectal toxicity following prostate cancer radiation therapy.* International Journal of Radiation Oncology* Biology* Physics, 2014. **89**(5): p. 1024-1031.

90. Sharma, M., S.K. Singh, P. Agrawal, and V. Madaan, *Classification of clinical dataset of cervical cancer using KNN.* Indian Journal of Science and Technology, 2016. **9**(28): p. 1-5.

91. Medjahed, S.A., T.A. Saadi, and A. Benyettou, *Breast cancer diagnosis by using k-nearest neighbor with different distances and classification rules.* International Journal of Computer Applications, 2013. **62**(1).

92. Li, C., et al., *Using the K-nearest neighbor algorithm for the classification of lymph node metastasis in gastric cancer.* Computational and mathematical methods in medicine, 2012. **2012**.

93. Htay, T.T. and S.S. Maung. *Early stage breast cancer detection system using glcm feature extraction and k-nearest neighbor (k-NN) on mammography image*. in *2018 18th International Symposium on Communications and Information Technologies (ISCIT)*. 2018. IEEE.

94. Murugan, T.D. and M.G. Kanojia. *Breast cancer detection using texture features and knn algorithm*. in *Hybrid Intelligent Systems: 20th International Conference on Hybrid Intelligent Systems (HIS 2020), December 14-16, 2020*. 2021. Springer.

95. Dubey, A.K., U. Gupta, and S. Jain, *Analysis of k-means clustering approach on the breast cancer Wisconsin dataset.* International journal of computer assisted radiology and surgery, 2016. **11**: p. 2033-2047.

96. Yadav, A.K., D. Tomar, and S. Agarwal. *Clustering of lung cancer data using foggy k-means*. in *2013 International Conference on Recent Trends in Information Technology (ICRTIT)*. 2013. IEEE.

97. Belciug, S., A.-B. Salem, F. Gorunescu, and M. Gorunescu. *Clustering-based approach for detecting breast cancer recurrence*. in *2010 10th International Conference on Intelligent Systems Design and Applications*. 2010. IEEE.

98. Liu, L. *Research on logistic regression algorithm of breast cancer diagnose data by machine learning*. in *2018 International Conference on Robots & Intelligent System (ICRIS)*. 2018. IEEE.

99. Tirzïte, M., et al., *Detection of lung cancer with electronic nose and logistic regression analysis.* Journal of breath research, 2018. **13**(1): p. 016006.

100. Seddik, A.F. and D.M. Shawky. *Logistic regression model for breast cancer automatic diagnosis*. in *2015 SAI Intelligent Systems Conference (IntelliSys)*. 2015. IEEE.

101. Salmi, N. and Z. Rustam. *Naïve Bayes classifier models for predicting the colon cancer*. in *IOP conference series: materials science and engineering*. 2019. IOP Publishing.

102. Zaw, H.T., N. Maneerat, and K.Y. Win. *Brain tumor detection based on Naïve Bayes Classification*. in *2019 5th International Conference on engineering, applied sciences and technology (ICEAST)*. 2019. IEEE.

103. Rashmi, G., A. Lekha, and N. Bawane. *Analysis of efficiency of classification and prediction algorithms (Naïve Bayes) for Breast Cancer dataset*. in *2015 International Conference on Emerging Research in Electronics, Computer Science and Technology (ICERECT)*. 2015. IEEE.

104. Rathi, M. and A.K. Singh, *Breast cancer prediction using Naïve Bayes classifier.* International Journal of Information Technology & Systems, 2012. **1**(2): p. 77-80.

105. Adi, K., et al., *Naïve Bayes algorithm for lung cancer diagnosis using image processing techniques.* Advanced Science Letters, 2017. **23**(3): p. 2296-2298.

106. Zhang, X., et al., *Identification of cancer-related long non-coding RNAs using XGBoost with high accuracy.* Frontiers in genetics, 2019. **10**: p. 735.

107. Yu, D., et al., *Copy number variation in plasma as a tool for lung cancer prediction using Extreme Gradient Boosting (XGBoost) classifier.* Thoracic cancer, 2020. **11**(1): p. 95-102.

108. Alfian, G., et al., *Predicting breast cancer from risk factors using SVM and extra-trees-based feature selection method.* Computers, 2022. **11**(9): p. 136.

109. Sathishkumar, R., K. Kalaiarasan, A. Prabhakaran, and M. Aravind. *Detection of lung cancer using SVM classifier and KNN algorithm*. in *2019 IEEE International Conference on System, Computation, Automation and Networking (ICSCAN)*. 2019. IEEE.

110. Sahu, B., S. Mohanty, and S. Rout, *A hybrid approach for breast cancer classification and diagnosis.* EAI Endorsed Transactions on Scalable Information Systems, 2019. **6**(20).

111. Vijila Rani, K. and S. Joseph Jawhar, *Lung lesion classification scheme using optimization techniques and hybrid (KNN-SVM) classifier.* IETE Journal of Research, 2022. **68**(2): p. 1485-1499.

112. Solanki, Y.S., et al., *A hybrid supervised machine learning classifier system for breast cancer prognosis using feature selection and data imbalance handling approaches.* Electronics, 2021. **10**(6): p. 699.

113. Zheng, B., S.W. Yoon, and S.S. Lam, *Breast cancer diagnosis based on feature extraction using a hybrid of K-means and support vector machine algorithms.* Expert Systems with Applications, 2014. **41**(4): p. 1476-1482.

114. Pirmoradi, S., M. Teshnehlab, N. Zarghami, and A. Sharifi, *A self-organizing deep neuro-fuzzy system approach for classification of kidney cancer subtypes using mirna genomics data.* Computer Methods and Programs in Biomedicine, 2021. **206**: p. 106132.

115. Johra, F.-T. and M.M.H. Shuvo. *Detection of breast cancer from histopathology image and classifying benign and malignant state using fuzzy logic*. in *2016 3rd International Conference on Electrical Engineering and Information Communication Technology (ICEEICT)*. 2016. IEEE.

116. Jindal, N., et al., *Fuzzy logic systems for diagnosis of renal cancer.* Applied Sciences, 2020. **10**(10): p. 3464.

117. Wang, C.-Y., et al., *Predicting survival of individual patients with esophageal cancer by adaptive neuro-fuzzy inference system approach.* Applied Soft Computing, 2015. **35**: p. 583-590.

118. Khalil, A.M., et al., *A new expert system in prediction of lung cancer disease based on fuzzy soft sets.* Soft Computing, 2020. **24**(18): p. 14179-14207.

119. Miranda, G.H.B. and J.C. Felipe, *Computer-aided diagnosis system based on fuzzy logic for breast cancer categorization.* Computers in biology and medicine, 2015. **64**: p. 334-346.

120. Goudarzi, M. and K. Maghooli, *Extraction of fuzzy rules at different concept levels related to image features of mammography for diagnosis of breast cancer.* Biocybernetics and Biomedical Engineering, 2018. **38**(4): p. 1004-1014.

121. Chatterjee, S. and A. Das, *A novel systematic approach to diagnose brain tumor using integrated type-II fuzzy logic and ANFIS (adaptive neuro-fuzzy inference system) model.* Soft Computing, 2020. **24**(15): p. 11731-11754.

122. Boadh, R., et al., *Study and prediction of prostate cancer using fuzzy inference system.* Materials Today: Proceedings, 2022. **56**: p. 157-164.

123. Nilashi, M., O. Ibrahim, H. Ahmadi, and L. Shahmoradi, *A knowledge-based system for breast cancer classification using fuzzy logic method.* Telematics and Informatics, 2017. **34**(4): p. 133-144.

124. Malibari, A.A., et al., *Optimal deep neural network-driven computer aided diagnosis model for skin cancer.* Computers and Electrical Engineering, 2022. **103**: p. 108318.

125. Bhardwaj, A. and A. Tiwari, *Breast cancer diagnosis using genetically optimized neural network model.* Expert Systems with Applications, 2015. **42**(10): p. 4611-4620.

126. Atban, F., E. Ekinci, and Z. Garip, *Traditional machine learning algorithms for breast cancer image classification with optimized deep features.* Biomedical Signal Processing and Control, 2023. **81**: p. 104534.

127. Sun, J., et al., *Five-Year Prognosis Model of Esophageal Cancer Based on Genetic Algorithm Improved Deep Neural Network.* IRBM, 2023. **44**(3): p. 100748.

128. Zorkafli, M.F., et al., *Classification of cervical cancer using hybrid multi-layered perceptron network trained by genetic algorithm.* Procedia Computer Science, 2019. **163**: p. 494-501.

129. Ding, L., X.-y. Zhang, D.-y. Wu, and M.-l. Liu, *Application of an extreme learning machine network with particle swarm optimization in syndrome classification of primary liver cancer.* Journal of Integrative Medicine, 2021. **19**(5): p. 395-407.

130. Sheikhpour, R., M.A. Sarram, and R. Sheikhpour, *Particle swarm optimization for bandwidth determination and feature selection of kernel density estimation based classifiers in diagnosis of breast cancer.* Applied Soft Computing, 2016. **40**: p. 113-131.

131. Thawkar, S., S. Sharma, M. Khanna, and L. kumar Singh, *Breast cancer prediction using a hybrid method based on Butterfly Optimization Algorithm and Ant Lion Optimizer.* Computers in Biology and Medicine, 2021. **139**: p. 104968.

132. Fekri-Ershad, S. and S. Ramakrishnan, *Cervical cancer diagnosis based on modified uniform local ternary patterns and feed forward multilayer network optimized by genetic algorithm.* Computers in Biology and Medicine, 2022. **144**: p. 105392.

133. Aslan, M.F., *A hybrid end-to-end learning approach for breast cancer diagnosis: convolutional recurrent network.* Computers and Electrical Engineering, 2023. **105**: p. 108562.

134. Ahmed, M. and M.R. Islam, *A combined feature-vector based multiple instance learning convolutional neural network in breast cancer classification from histopathological images.* Biomedical Signal Processing and Control, 2023. **84**: p. 104775.

135. Tyagi, S. and S.N. Talbar, *LCSCNet: A multi-level approach for lung cancer stage classification using 3D dense convolutional neural networks with concurrent squeeze-and-excitation module.* Biomedical Signal Processing and Control, 2023. **80**: p. 104391.

136. Wang, Y., et al., *Multimodal convolutional neural networks based on the Raman spectra of serum and clinical features for the early diagnosis of prostate cancer.* Spectrochimica Acta Part A: Molecular and Biomolecular Spectroscopy, 2023. **293**: p. 122426.

137. Sánchez-Cauce, R., J. Pérez-Martín, and M. Luque, *Multi-input convolutional neural network for breast cancer detection using thermal images and clinical data.* Computer Methods and Programs in Biomedicine, 2021. **204**: p. 106045.

138. Shamshiri, M.A., A. Krzyżak, M. Kowal, and J. Korbicz, *Compatible-domain Transfer Learning for Breast Cancer Classification with Limited Annotated Data.* Computers in Biology and Medicine, 2023. **154**: p. 106575.

139. Aljuaid, H., et al., *Computer-aided diagnosis for breast cancer classification using deep neural networks and transfer learning.* Computer Methods and Programs in Biomedicine, 2022. **223**: p. 106951.

140. Bratchenko, I.A., et al., *Classification of skin cancer using convolutional neural networks analysis of Raman spectra.* Computer Methods and Programs in Biomedicine, 2022. **219**: p. 106755.

141. Ghoneim, A., G. Muhammad, and M.S. Hossain, *Cervical cancer classification using convolutional neural networks and extreme learning machines.* Future Generation Computer Systems, 2020. **102**: p. 643-649.

142. Rahman, M.M., M.S.I. Khan, and H.M.H. Babu, *BreastMultiNet: A multi-scale feature fusion method using deep neural network to detect breast cancer.* Array, 2022. **16**: p. 100256.

143. Sun, W., T.-L.B. Tseng, J. Zhang, and W. Qian, *Enhancing deep convolutional neural network scheme for breast cancer diagnosis with unlabeled data.* Computerized Medical Imaging and Graphics, 2017. **57**: p. 4-9.

144. Yang, Z., et al., *EMS-Net: Ensemble of multiscale convolutional neural networks for classification of breast cancer histology images.* Neurocomputing, 2019. **366**: p. 46-53.

145. Deb, S.D. and R.K. Jha, *Breast UltraSound Image classification using fuzzy-rank-based ensemble network.* Biomedical Signal Processing and Control, 2023. **85**: p. 104871.

146. Yang, X., et al., *Triple-attention interaction network for breast tumor classification based on multi-modality images.* Pattern Recognition, 2023. **139**: p. 109526.

147. Chaudhury, S. and K. Sau, *A BERT encoding with Recurrent Neural Network and Long-Short Term Memory for breast cancer image classification.* Decision Analytics Journal, 2023. **6**: p. 100177.

148. Krenzer, A., et al., *A real-time polyp-detection system with clinical application in colonoscopy using deep convolutional neural networks.* Journal of Imaging, 2023. **9**(2): p. 26.

149. Walczak, S. and V. Velanovich, *Improving prognosis and reducing decision regret for pancreatic cancer treatment using artificial neural networks.* Decision Support Systems, 2018. **106**: p. 110-118.

150. Bhambhvani, H.P., et al. *Development of robust artificial neural networks for prediction of 5-year survival in bladder cancer*. in *Urologic Oncology: Seminars and Original Investigations*. 2021. Elsevier.

151. Fu, J., et al., *Development and validation of a predictive model for peripherally inserted central catheter-related thrombosis in breast cancer patients based on artificial neural network: A prospective cohort study.* International Journal of Nursing Studies, 2022. **135**: p. 104341.

152. de Souza, N.M.P., et al., *Discrimination of molecular subtypes of breast cancer with ATR-FTIR spectroscopy in blood plasma coupled with partial least square-artificial neural network discriminant analysis (PLS-ANNDA).* Chemometrics and Intelligent Laboratory Systems, 2023: p. 104826.
